# Supplementary material for: The Summer North Atlantic Oscillation, Arctic sea ice, and Arctic jet Rossby wave forcing
Source: Sci Adv. 2024 Nov 13;10(46):eadk6693. doi: 10.1126/sciadv.adk6693 (PMC11639673; doi:10.1126/sciadv.adk6693)
Supplement: 20241113-1 [file sciadv.adk6693.v1.pdf]

CLIMATOLOGY

The Summer North Atlantic Oscillation, Arctic sea ice, and Arctic jet Rossby wave forcing

Chris K. Folland[1,2,3,4]*, Tinghai Ou[3], Hans W. Linderholm[3], Adam A. Scaife[1,5], Jeff Knight[1], Deliang Chen[3]

Copyright © 2024 the authors; exclusive licensee American Association for the Advancement of Science subject to: British Crown copyright, © British Crown Copyright, 2024, Met Office. Distributed under a Creative Commons Attribution License 4.0 (CC BY).

We use Coupled Model Intercomparison Project Phase 6 (CMIP6) coupled and Atmospheric Model Intercomparison Project (AMIP) climate models, dynamical analyses, and observations to investigate interactions between summer Arctic sea ice concentration (SIC) variations and the Summer North Atlantic Oscillation (SNAO). Observations suggest that SIC-SNAO relationships mainly come from the East Siberian to Arctic Canada (ESAC) region where a weak atmospheric jet stream exists in summer. Twelve CMIP6 models with the most realistic atmospheric climatologies over the North Atlantic and Europe agree well with reanalyses on relationships between SIC and Northern Hemisphere atmospheric circulation. CMIP6 model data indicate that ESAC SIC influences the SNAO with a lead time of several weeks. However, AMIP simulations do not reproduce the observed atmospheric circulation when observed sea ice is prescribed. Rossby wave analyses do though support observed ESAC SIC influences on the SNAO. We conclude that ESAC Arctic SIC modestly influences the SNAO, and such investigations require the use of coupled models.

INTRODUCTION

The Arctic has experienced substantial warming in recent decades, several times more than the global average (*1*, *2*), accompanied by a substantial decrease in the areal extent and by thinning of the sea ice (*3*–*5*). Thus, since the late 1980s, the globe has warmed by about 0.6°C, while the Arctic has warmed nearly 2.5°C (*2*). There have been particularly strong losses of areal extent in late summer and autumn to successive record low levels in 2007 and 2012, while 2020 has shown the second lowest minimum September levels, up to 2023, since 1979. Melting Arctic sea ice influences weather and climate locally in the Arctic but may also have a remote impact on the Northern Hemisphere (NH) mid-latitudes by perturbing local energy fluxes at the surface and modifying the atmospheric and oceanic circulation (*6*–*9*). Particularly in the cold season, enhanced heat fluxes from a more open and warmer Arctic Ocean have led to considerable warming over the polar region (*1*). It has been argued that these surface changes can induce anticyclonic atmospheric circulation anomalies over the Arctic, similar to those observed during the negative phase of the North Atlantic Oscillation (NAO) or Arctic Oscillation (*10*, *11*), although other responses are seen in different models (*12*–*14*). Moreover, observational as well as model studies have suggested influences of these anomalously enhanced surface heat and moisture fluxes on regional mid-latitude weather (*6*, *9*, *15*–*18*). This atmospheric impact during the cold season relates to the peak in surface flux that occurs in late autumn and early winter because of the large ocean-atmosphere temperature gradient (*19*). Recently, controlled experiments from multiple climate models indicate that Arctic sea ice indeed perturbs winter NH atmospheric circulation, though the effects are quite weak (*20*). During summer, however, Arctic sea ice is more affected by near-surface winds and incoming solar radiation, suggesting a strong atmospheric driver of year-to-year summer sea ice variability (*21*, *22*).

Although any atmospheric circulation response to reduced sea ice in the Arctic during summer may be smaller in magnitude than in winter, reduced natural variability during summer facilitates potentially larger signal-to-noise ratios and detection of atmospheric circulation changes (*23*). Several studies focusing on summer, using both observational and climate model data, have proposed links between Arctic sea ice extent (SIE) or sea ice concentration (SIC) and atmospheric circulation (*23*–*26*). Others (*27*, *28*) also point to sea ice effects on the atmosphere in summer including how summer climate over Northwest Europe could be influenced by spring sea ice variations in both the Sea of Okhotsk and the Barents-Kara Sea (*29*). Evidence has also been found that varying winter SIC conditions west of Greenland may have influenced the subsequent summer atmospheric circulation over northern Eurasia (*30*) and that a link may exist between spring Arctic SIC conditions and the summer monsoon circulation over East Asia (*31*). In addition, with the help of an atmosphere only climate model, it was found that sea ice loss, together with increased sea surface temperature (SST) in the Labrador Sea affects the summer atmospheric circulation over the North Atlantic region (*24*). Similar results were also obtained using a fully coupled climate model (*26*). On the other hand, several studies point to the tropics as a source of atmospheric circulation anomalies that affect sea ice (*32*–*34*)· A further finding is that reduced Arctic sea ice causes low pressure anomalies over the polar region and subsequently triggers south-eastward Rossby wave trains propagating from northern Europe to East Asia (*35*). This induces an anomalous anticyclone over Southwestern China, leading to increased summer heatwave frequency. On the basis of observational analyses and numerical experiments, it has also been suggested that the interdecadal increase in European summer heat waves is linked to the reductions in SIC and Eurasian snow cover fraction across the mid-high latitudes (*36*). Last, an appreciable difference in summer temperature and heatwave frequency has been associated with anomalous summer sea ice melting between the mid- and high latitudes of Asia, North America and Europe (*37*). Together, these studies suggest potential impacts of melting Arctic sea ice on the mid-latitude atmospheric circulation in summer.

[1]Met Office, Exeter, Devon, UK. [2]University of East Anglia, Norwich, UK. [3]University of Gothenburg, Gothenburg, Sweden. [4]University of Southern Queensland, Toowoomba, Australia. [5]Department of Mathematics, University of Exeter, Exeter, UK.
*Corresponding author. Email: chris.folland@metoffice.gov.uk

One limitation of existing summer sea ice studies is that it is difficult to determine cause and effect as atmospheric circulation and sea ice effects may be almost simultaneous (*38*). There is little doubt that summer atmospheric circulation quite strongly affects summer sea ice (*21*, *39*). This does not of course preclude a coupled relationship where the summer atmospheric circulation influences change the pattern of summer sea ice which in turn affects the overlying atmosphere and perhaps the more remote atmosphere and ocean surface. If so, coupled models will be essential for sea ice–climate studies, although it still may be difficult to fully unravel the details of cause and effect.

The chief, though not sole, focus of this paper is on the summer climate of the North Atlantic and Europe and its interactions with Arctic sea ice variations and the Arctic atmosphere. The most important atmospheric pattern to influence these regions is the Summer North Atlantic Oscillation (SNAO) (*40*), which we concentrate on. Most prominent during winter, the NAO is one of the few modes of climate variability that persists throughout the year, though details of its pattern change seasonally (*41*). Accordingly, the positive and negative nodes of the dipole NAO pattern have more northerly positions during summer, and the dipole pattern has a somewhat smaller spatial scale. During summer, the NAO pattern has pressure centers located over the British Isles/Scandinavia and over Greenland (*40*). The SNAO, defined in this paper as the average for June, July, and August or its subseasonal periods down to a single month (see Materials and Methods and Results), is strongly related to changes in North Atlantic and European summer storm tracks (*40*, *42*). In its negative phase, the SNAO is associated with cyclonic conditions over northwest Europe from UK to Scandinavia, yielding wet conditions there and corresponding anticyclonic conditions centered over Greenland. By contrast, a strong positive phase of the SNAO is related to summer droughts over northwest Europe, from the UK to Scandinavia in particular (*43*) and a northerly position of the main storm track. In the negative SNAO phase, the storm track moves ~10° latitude south, giving cloudy, wet, and cooler conditions over the same region. After having been mainly in a positive phase since the late 1960s, after 2006, the SNAO entered a mainly negative phase (*44*, *45*), only 2013, 2018, and 2022 being distinctly positive since (up to and including 2023). This tendency coincided with an accelerated melting of Arctic sea ice (*4*), and it has been suggested that these high-precipitation northern European summers could possibly be linked, at least in part, to regional changes in Arctic SIC especially in summer (*23*).

The response of the NH atmosphere to sea ice changes is complicated by internal atmospheric variability that may be independent of SIE variations or driven by other factors such as El Niño (*34*, *40*, *46*) and SST influences more generally (*47*). Thus, worldwide SST variations in models could be connected to sea ice influences on climate, a problem complicated by the different responses of climate models to sea ice changes. Here, we concentrate on the relationship between the SNAO, related NH circulation conditions, and sea ice extent in summer. We use a recent HadISST2 dataset of SIC (*48*), the European Centre for Medium Range Weather Forecasts version 5 (ERA5) reanalysis dataset (*49*) and a multimodel approach using Coupled Model Intercomparison Project Phase 6 (CMIP6) model simulations (*50*) over the period 1979–2015. However, we first explore immediately prior late-spring sea ice relationships with the SNAO. We identify Arctic Ocean regions likely to be most strongly linked to the SNAO using a correlation analysis between the observed SNAO and observed SIC checked using a maximum covariance analysis between SIC and observed pressure at mean sea level (PMSL) in the North Atlantic/European region. This procedure makes no prior assumptions about the existence of the SNAO or the direction of any influences. We select CMIP6 models likely to be the most useful by choosing those with the most realistic atmospheric climatology over the North Atlantic European region. We then use daily CMIP6 data from this set of models to show the intraseasonal response of the SNAO to SIC changes and those of SIC to the SNAO in the SIC region identified as best related to the SNAO. We examine how the NH atmospheric circulation may be involved in these interactions, including the role of Rossby waves. Last, we test how important the role of SIC may be in influencing the SNAO by using CMIP6 generation atmospheric only [Atmospheric Model Intercomparison Project (AMIP)] models to investigate whether the coupled model results are reproduced in models with prescribed observed SIC.

RESULTS

The SNAO

We extend the existing definition of the SNAO (*40*) to include the complete Arctic in the North Atlantic sector (80°W to 40°E, 35°N to 90°N) and the whole summer June–August (JJA), as well as for subseasonal periods within summer. This can now be done reliably as Arctic reanalyses have improved since the original publication of the SNAO pattern (*40*) and it is important to better represent the SNAO node over Greenland. Figure 1A shows the JJA SNAO pattern, defined here for the well observed period 1951–2010 as the first covariance eigenvector [empirical orthogonal function 1 (EOF1)] of PMSL over this region (see also Materials and Methods). Quite similar patterns for JJA, for July and August (JA) and August alone are shown in Fig. 1 (B and C). These patterns include the Greenland region. A consequence of fully including Greenland is that the percentage of PMSL variance explained by the SNAO is considerably greater than reported before (*40*), being 41.5% in 1951–2010 for JJA and 37.5% for JA. This JJA percentage now approaches that for the winter NAO in December–February. Even the SNAO pattern for August alone reaches a variance explained of 34.1% for this period. Compared to the daily, 10-day, and JA periods originally described (*40*), the SNAO patterns calculated here for JJA, JA, and August are similar. We have though retained separate patterns for these three periods as some relatively minor differences between the June pattern and the combined JApattern were noted before (*40*).

Observed summer SIC associations with the SNAO

The correlation between the SNAO and late-spring SIC throughout the Arctic was first tested for 1979–2015 and then the longer period 1961–2019, using April–May and May-only SIC. No statistically significant overall correlations were found, field significance being only at the 20% level (1961–2019) in May and lower than 20% in April–May. Thus, spring Arctic sea ice is not statistically significantly related to the SNAO in JJA. Observed correlations of the SNAO with contemporaneous Arctic SICs are shown in Fig. 2 (A and B) between the SNAO and SIC (see also Materials and Methods) for 1979–2015 in JJA, JA, and August, respectively. The strongest correlations are, as expected, for the longest summer period used, JJA (Fig. 2A), as synoptic weather noise is more effectively averaged out. Statistically significant positive correlations in the Canadian/Alaskan/East Siberian region imply that high (low) SIC there is positively

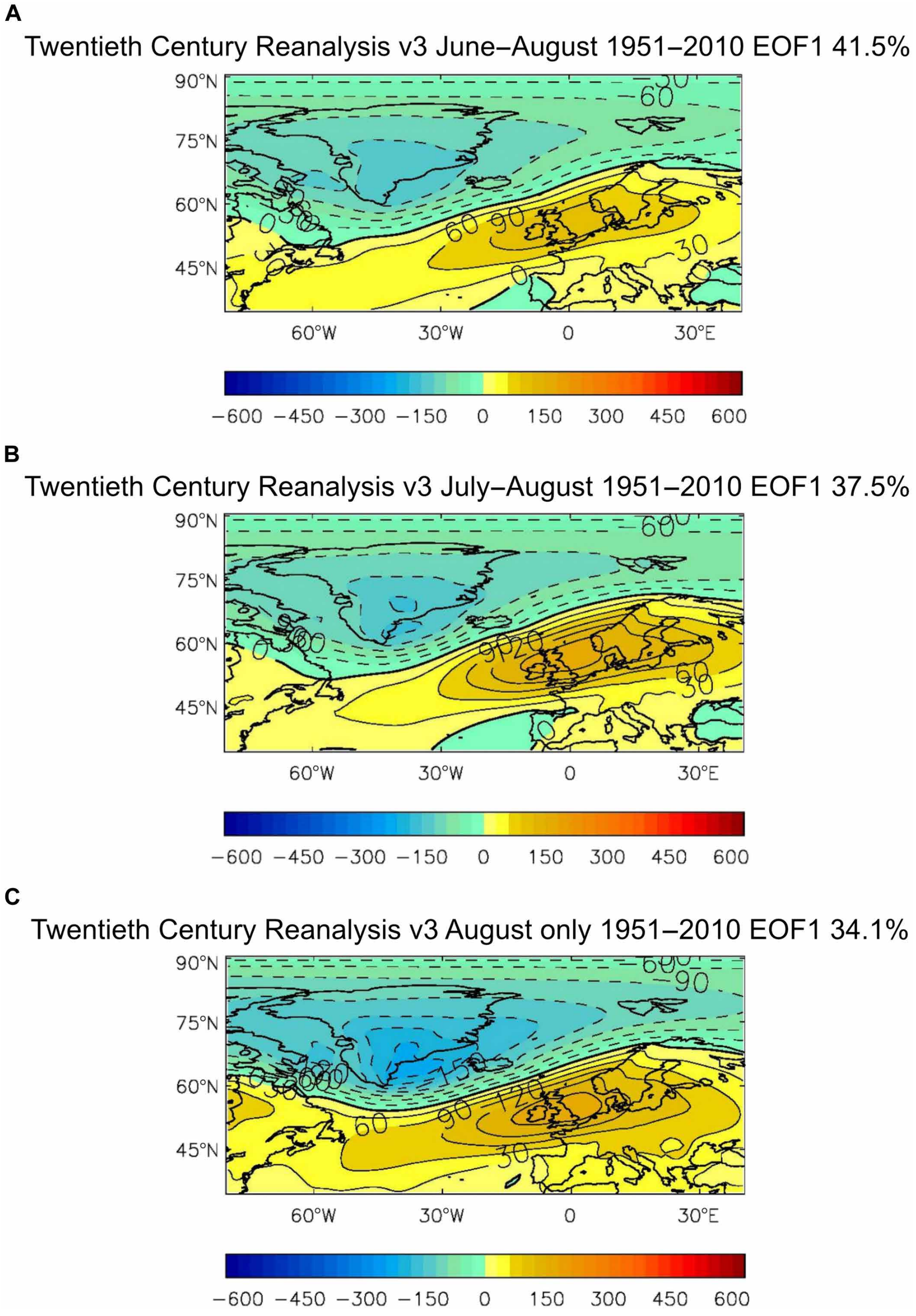

Fig. 1. Patterns of the SNAO. These are the first covariance eigenvectors (EOF1) of PMSL for the North Atlantic–European region 80°W to 40°E, 35°N to 90°N. (**A**) For June–July-August (JJA), (**B**) for July–August (JA), and (**C**) for August (Aug) for 1951–2010 for the positive SNAO using the 20th century reanalysis PMSL data version 3 (*86*). Dashed contours and blue colors are negative while filled contours and yellow and brown colors are positive.

correlated with a high (low) SNAO. We call this the East Siberian to Arctic Canada region (ESAC) region of the Arctic Ocean.

This region is chosen to be 55°N to 81°N, 55°W to 150°E in the analyses described below and includes ocean regions immediately west of Greenland but excludes other relatively nearby areas, including those outside the Arctic like the Sea of Okhotsk, where summer sea ice is mostly absent. The JJA correlation pattern has an Arctic Ocean (≥ 65°N) field significance (*51*) <0.1%, indicating that this pattern is highly statistically significant overall, though this on its own does not identify the direction of influence. Figure 2B for JA is similar to JJA, also field significant at <0.1% level. The much weaker, but broadly similar, correlation pattern in August is just field significant at the 5% level. All three periods also show locally statistically significant correlation areas outside the ESAC region, mainly

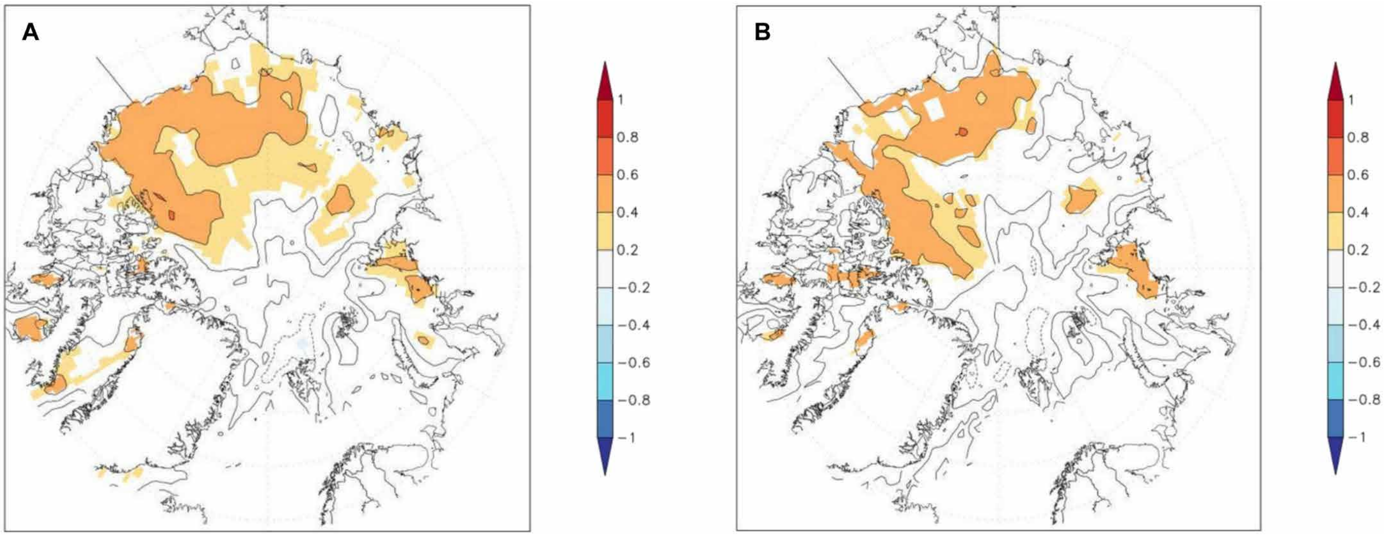

Fig. 2. Correlations between the SNAO and observed SICs for 1979–2015. (**A**) For JJA. (**B**) For JA. Areas with correlations locally significant using a two-sided *t* test at the 5% level are shown in color. The SNAO series have insignificant first serial correlations over 1979–2015 so no persistence corrections are needed for estimating correlation significance. The period is chosen to be the same as used below for the CMIP6 model analyses.

near the Taymyr Peninsula in western Siberia, so there could well be more limited SIC relationships with the SNAO from outside the ESAC region. It is worth noting that May ESAC SIC is significantly correlated at the 2% level at 0.64 with JJA ESAC SIC over the reasonably reliable period 1961–2019, correlation being enhanced by a similar trend, so that the interannual correlation is insignificant at only 0.19. Note that usable Arctic SIC data starts around 1953 [(*48*); see their figure 10], but we start at 1961 for convenience. May ESAC SIC is also poorly and insignificantly correlated with the JJA SNAO at 0.22, so we do not consider spring ESAC SIC further.

A different method of identifying these relationships that makes no a priori assumptions about the existence of the SNAO, and so strengthens the case for any relationships, is to use maximum covariance analysis, sometimes called singular value decomposition or SVD (see Materials and Methods). Figure 3 (A to F) shows the pair of SVD patterns explaining most covariance of simultaneous Arctic Ocean SIC and PMSL in the North Atlantic/European region for each of JJA, JA, and August. The three SIC patterns all mirror the correlation diagrams described in Fig. 2 with relatively minor differences between summer periods. The most prominent PMSL patterns for each period are all quite similar to the SNAO patterns in Fig. 1, though with less emphasis on Scandinavia and more over the UK and to its west.

Table S1 shows the covariance squared explained by the first and second SVD modes identified in JJA, JA, and August (SVD1 and SVD2). SVD1 (the SNAO-like mode) explains much more of the squared covariance than SVD2 for all three periods, reaching 42% in JJA with SVD2 only explaining just over 10%. In JA, these percentages are 37 and 11%, while in August, the first pattern explains just over 28% with SVD2 explaining 10% again. This implies that the dominant relationship between Arctic SIC changes and summer North Atlantic PMSL is indeed described by the SNAO. In summary, the SVD analysis strengthens the case for the correlation-based associations between the SNAO and Arctic SIC shown in Fig. 2 (A and B). However, relationships between the SNAO and regional Arctic sea ice could well be nonstationary in the longer term. This has been demonstrated for relationships between October Barents-Kara Sea ice variability and the winter NAO in observations and models (*52*).

Figure 3G shows the time series of the standardized JJA SNAO and SIC in the ESAC region for the longer reasonably well-observed SIC period 1961–2019. JA and August are broadly similar. (Figure S1 shows, for completeness, the absolute time-varying fractions of SIC in the ESAC region for JJA, JA, and August. We also show the time series in Fig. 3G of what we term the regional SNAO for JJA. Like some versions of the well-known winter NAO indices, this is an index of PMSL differences between two geographical regions, here the difference between the region [50°N to 60°N, 5°W to 15°E], near the center of the southern SNAO mode in the North Sea, and the region [60°N to 75°N, 30°W to 55°W], near the center of the northern, Greenland, node. The regional SNAO indices behave similarly to the SNAO index based on the EOF patterns in Fig. 1 (A and B) in both JJA and JA, correlations being 0.97 (JJA) and 0.96 (JA) over 1979–2015 and are used below when climate models are analyzed (see Materials and Methods). The August Fig. 1C and August regional SNAO indices are also quite similar with a correlation of 0.89 over the same period. Table 1 (top) summarizes the correlations between the ESAC SIC time series and the JJA and JA SNAO time series over 1979–2015. All correlations are significant at the 5% level or better. For our longer period 1961–2019, the correlations with ESAC SIC are lower but still all significant at the 5% level.

Over 1979–2015, the JJA SNAO EOF1 has a correlation of 0.55 with ESAC SIC in JJA and the regional index has a similar correlation of 0.52, suggesting that at least 25% of the variance of the SNAO links to SIC variations over this period. Furthermore, the correlation of the corresponding JJA SNAO indices with JA SIC is slightly higher at 0.57 and 0.55, respectively, not unexpected as the main variations in sea ice have been in JA. Over the longer period 1961–2019, these values are only slightly lower.

Figure 3H shows homogeneous time series of the pairs of PMSL (with a negative sign) and SIC SVD1 patterns for JJA, standardized over 1979–2015. The JA and August SIC and negative of the PMSL vector time series are also similar (not shown). This indicates that

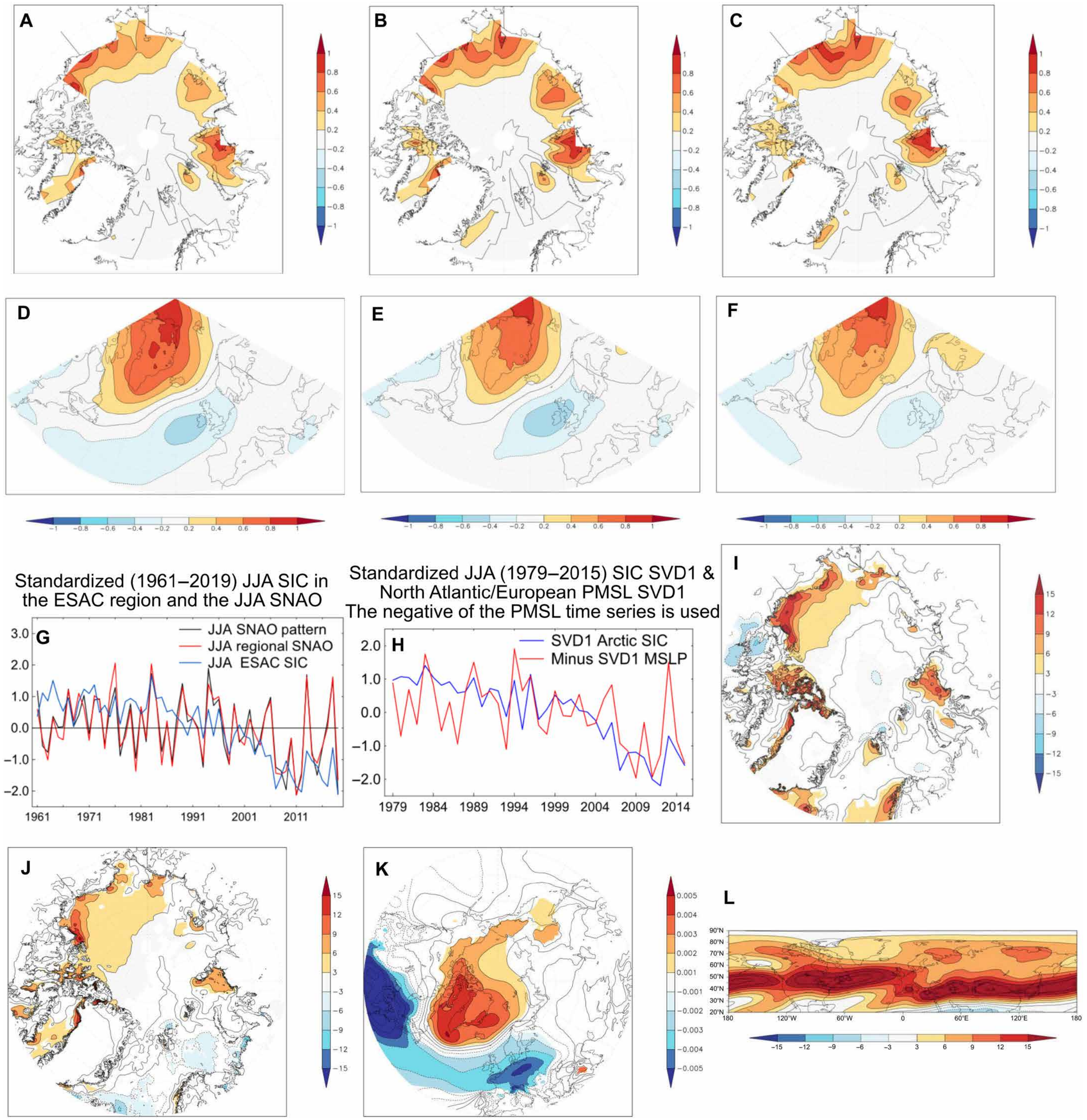

Fig. 3. Observed relationships between the SNAO and various diagnostics of the Arctic climate. (**A** to **F**) Results of a maximum covariance analysis between Arctic SIC and the SNAO for 1979–2015, in which (A) to (C) are SIC vectors throughout the Arctic Ocean for JJA, JA, and Aug, respectively, explaining the most variance using the first mode of this analysis; (D) to (F) are PMSL vectors in the North Atlantic-European region defined for the SNAO for JJA, JA, and Aug, respectively. (**G**) Standardized time series of the JJA SNAO pattern and its regional index version together with ESAC, 1961–2019. (**H**) The time series of the first mode of the singular value decomposition (SVD1) in JJA for Arctic SIC and the negative of the time series for PMSL SVD1 in the North Atlantic-European SNAO region. (**I**) Regression of JJA net surface radiation >65°N on the standardized negative JJA SNAO (SD) 1979–2015, the period of the model results, wm-^{2}SD^{-1}. (**J**) Regression of JJA net surface upwards hear flux >65°N on the standardized negative JJA SNAO (SD) 1979–2015, the period of the model results, wm-^{2}SD^{-1}. (**K**) Regression of the standardized negative JJA SNAO 1951–2010, the period for calculating the SNAO pattern in Fig. 1, on JJA PMSL everywhere. >35°N, SDhPa^{-1}. JA and August are similar. (**L**) Average 300-hPa mean westerly flow (ms^{-1}) over the NH in JJA. On all maps except (L), only colored areas are locally significant at the 5% level.

the same basic covarying phenomena are being seen throughout summer. Table 1 (bottom) shows correlations between the time series of all the vectors where the sign of the left (SIC) vectors has been reversed. All correlations between left and right vectors for a given summer period are significant at the 5% level and have similar values at 0.55 (JJA), 0.54 (JA), and 0.55 (August), August values being larger than those between the August SNAO and the ESAC indices at 0.38 (still significant at the 5% level). This enhanced correlation may relate to the westward shift in the August PMSL right vector relative to the EOF1 SNAO pattern.

We next explore to what extent a link exists between the JJA SNAO and surface net solar and long-wave radiation that may influence sea ice over the Arctic Ocean. Figure 3I shows a regression of the negative standardized JJA SNAO (SD) against net surface radiation given by ERA5 for 1979–2015 where regions significant at better than the 5% level are colored. Figure 3I shows that the ESAC region is where these variables are most strongly linked over an extensive area, with values widely exceeding 10 wm^{-2} SD^{-1} between northwest Greenland and close to Alaska. Figure 3I therefore shows that there is quite a strong link between the negative JJA SNAO and ocean or ice net surface heating of most of the ESAC region with weaker or much more localized areas elsewhere in the Arctic. This is supported by the similar picture provided by Fig. 3J showing a regression of JJA net surface upwards hear flux against the strength of the negative SNAO. There is a statistically significant relationship over the whole ESAC region, exceeding 9 wm^2 SD^{-1} near the North American coast. This is supported by Fig. 3K, which shows that there is an almost Arctic wide influence of the SNAO on PMSL in JJA, strongest and most statistically significant in the ESAC region. Thus, when PMSL is high over Greenland during the negative phase of the SNAO, PMSL also tends to be statistically significantly higher than normal over much of the Arctic Ocean and especially the ESAC region. This is consistent with the net surface radiation regression pattern (Fig. 3I). Given these results, in the remainder of the paper, we concentrate on SIC variations in the ESAC region. There may also be other related SNAO influences on ESAC sea ice due to other atmospheric circulation influences, but these are beyond the scope of this paper.

Is there a summer atmospheric jet stream over the ESAC region?

Figure 3 suggests that the SNAO might, in part, originate over the ESAC region, e.g., by downstream Rossby wave propagation for which atmospheric westerlies would be needed locally. Figure 3L, for JJA 1979–2015 (JA and August are similar), shows that there is indeed a weak but distinct climatological Arctic westerly jet at 300 hPa over the ESAC region, considerably further north than the main mid-latitude westerly jets and slightly stronger in August than in JJA. There is also evidence elsewhere near the Arctic Ocean coast of weakly enhanced 300-hPa westerly flow. However, the ESAC summer or August Arctic jet stream varies considerably interannually. We next explore further how the SNAO is linked with SIC variations in the ESAC region and whether there is possible Rossby wave forcing of the SNAO itself.

Comparing CMIP6 models with observations

The period 1979–2015 was selected as 1979 was the first full year when satellite data became fully available for sea ice observations and hence when SIC was best observed (*48*). In addition, this was a period when positive and negative SIC extremes were particularly prominent. The observed atmospheric data were taken primarily from the ERA5 reanalysis (*49*) but also from the JRA-55 Reanalysis (*53*) and the National Centers for Environmental Prediction (NCEP)–Department of Energy (DOE) AMIP-II Reanalysis (R-2) (*54*) (hereafter NCEP2) (see Materials and Methods). The latter are only used (see below) in the multidaily analyses of leads and lags in the SNAO and SIC; otherwise, we use both ERA5 and JRA-55. The coupled CMIP6 models (*50*) were chosen to be the 12 having the best PMSL climatology in the North Atlantic/European region over the climatological 30-year period 1981–2010, so all these years are within our analysis period (see Materials and Methods). We use coupled models rather than atmosphere only

Table 1. Correlations coefficients between the SNAO and ESAC time series in June–July-August (JJA) and July–August (JA) and the SVD1 pattern time series in JJA, JA, and August using the negative of the SIC SVD1 vectors during 1979–2015.

	JJA SNAO	JJA SNAO REGIONAL	JA SNAO	JA SNAO REGIONAL	JJA ESAC SIC	JA ESAC SIC
JJA SNAO	1.00					
JJA SNAO REGIONAL	0.97	1.00				
JA SNAO	0.88	0.88	1.00			
JA SNAO REGIONAL	0.83	0.87	0.96	1.00		
JJA ESAC SIC	0.55	0.52	0.48	0.47	1.00	
JA ESAC SIC	0.57	0.55	0.50	0.49	0.99	1.00
	**SVD1 SIC JJA**	**SVD1 SIC JA**	**SVD1 SIC AUG**	**SVD1 MSLP JJA**	**SVD1 MSLP JA**	**SVD1 MSLP AUG**
SVD1 SIC JJA	1.00					
SVD1 SIC JA	0.997	1.00				
SVDI SIC AUG	0.994	0.997	1.00			
SVD1 MSLP JJA	0.55	0.53	0.54	1.00		
SVD1 MSLP JA	0.56	0.54	0.55	0.996	1.00	
SVD1 MSLP AUG	0.56	0.54	0.55	0.998	0.996	1.00

models for our main model investigation (but see below for a comparison with atmospheric models) as contemporaneous coupled interactions with the land and ocean surface inside and well outside the Arctic, including perhaps some parts of the tropics, are very likely to be important (*27*, *55*) for identifying an atmospheric signal from regional Arctic SIC variations. Table 2 (left) shows the 12 models having between one and eight ensemble members each, each ensemble member being given equal weight. Model results are for JJA, JA, and August. There are 31 ensemble members for the JJA, JA, and August mean analyses and 28 for the multidaily analysis. The regional SNAO index is used for both CMIP6 and the ERA5 and JRA-55 reanalyses as this provides a consistent index independent of different versions of the SNAO that may be created when using EOF analysis on different models (see Materials and Methods).

We first compare differences in SIC and several atmospheric variables over the NH in JJA, JA, and August for the 8 years that have the highest SIC in the ESAC region over the period 1979–2015 for JJA (1979, 1980, 1982, 1983, 1984, 1985, 1992, and 1996), and the 8 years with lowest concentrations over JJA (2007, 2008, 2009, 2010, 2011, 2012, 2014, and 2015). Eight years was chosen to maximize sample size without weakening the signal and represent approximately the top and bottom quintiles of ESAC SIC. Because there are 31 ensemble members each having 8 years for a given extreme, there are 248 maps for a given extreme. Tests using the five highest and lowest years gave similar results but with slightly lower significance due to the smaller sample size. Because we use the same years for all three summer periods, we might not always sample the 16 most extreme years for these shorter periods, but this likely only has a small effect. We use individual model ESAC SIC extremes so that observed and modeled SIC extremes will generally be in different years in different models.

Figure 4A shows results for the eight pairs of lowest minus highest SIC years over 1979–2015 in JJA, while fig. S2 shows similar results for JA and August. We first discuss SIC differences, noting that JJA, JA, and August are quite similar. Model and observation (HadISST2) differences between the two sets of extremes are broadly similar in all three summer periods. Consistent in both models and observations is some reduction in SIC west of north Greenland, included in the definition of the ESAC region. The most important finding is that models and observations (both reanalyses) agree quite well on the global pattern of PMSL differences between very low and high ESAC SIC years in Fig. 4A, but the observed increase of PMSL occurs more strongly across the high Arctic. A strong and statistically significant high PMSL region is seen over and around Greenland in both observations and models in JJA, surrounded at lower latitudes by widespread regions of statistically significantly lower PMSL in both models and observations. Negative PMSL is strong over the UK region in both models and observations, consistent with a strong projection of this part of the pattern associated with low ESAC SIC onto the modeled and observed negative SNAO. This large-scale pattern is also seen in the models and observations in JA and August (fig. S2) and also projects strongly onto the negative SNAO (Fig. 1, B and C). A strong projection in both models and observations onto the negative SNAO is consistent with studies using individual models (*23*, *26*, *28*). A further widely distributed but mostly weak high PMSL region in extreme low SIC years relative to high ones appears over Asia, extending into the Pacific in the models. This occurs in all three summer periods but is only sometimes locally statistically significant. Last, statistically

Table 2. The 12 CMIP6 and AMIP models chosen for having the PMSL statistics in the North Atlantic/European region 25°N to 90°N. 80°W to 40°E in 1981–2010 and the 10 CMIP6 models used for the multidaily analyses.

	CMIP6				AMIP		
**Rank**	**Model name**	**Model atmospheric resolution, degrees lat. × degrees long.**	**No. of ensemble members in JJA, JA, and August mean analyses**	**No. of ensemble members used in multidaily analyses**	**Model name**	**Model atmospheric resolution, degrees lat. × degrees long.**	**No. of ensemble members in JJA, JA, and August mean analyses**
1	AWI-CM-1-1-MR	0.935 × 0.938	1	0	CNRM-CM6-1-HR	0.499 × 0.500	1
2	EC-Earth3-Veg-LR	1.121 × 1.125	3	3	MPI-ESM1-2-HR	0.935 × 0.938	3
3	BCC-CSM2-MR	1.121 × 1.125	1	1	CIESM	1.250 × 0.942	1
4	EC-Earth3-Veg	0.702 × 0.703	8	8	EC-Earth3-Veg	0.703 × 0.702	1
5	EC-Earth3-CC	0.702 × 0.703	1	1	EC-Earth3	0.703 × 0.702	3
6	CAMS-CSM1-0	1.121 × 1.125	2	0	CAMS-CSM1-0	1.121 × 1.125	3
7	MRI-ESM2-0	1.121 × 1.125	5	5	GFDL-ESM4	1.250 × 1.000	1
8	CESM2	0.942 × 1.250	3	3	BCC-ESM1	2.813 × 2.791	3
9	MPI-ESM1-2-HR	0.935 × 0.938	2	2	HadGEM3-GC31-MM	0.833 × 0.556	4
10	CESM2-WACCM	0.942 × 1.250	3	3	MPI-ESM1-2-LR	1.875 × 1.865	3
11	GFDL-CM4	1.000 × 1.250	1	1	FGOALS-f3-L	1.250 × 1.000	2
12	CNRM-CM6-1-HR	0.499 × 0.500	1	1*	GISS-E2-2-G	2.500 × 2.000	4

*The starred CMIP6 value at Rank 12 is due to the daily sea ice fraction being not available for the year 2015 under warming scenario SSP245; accordingly, the result for both daily PMSL and sea ice fraction under scenario SSP 285 is used for the year 2015.

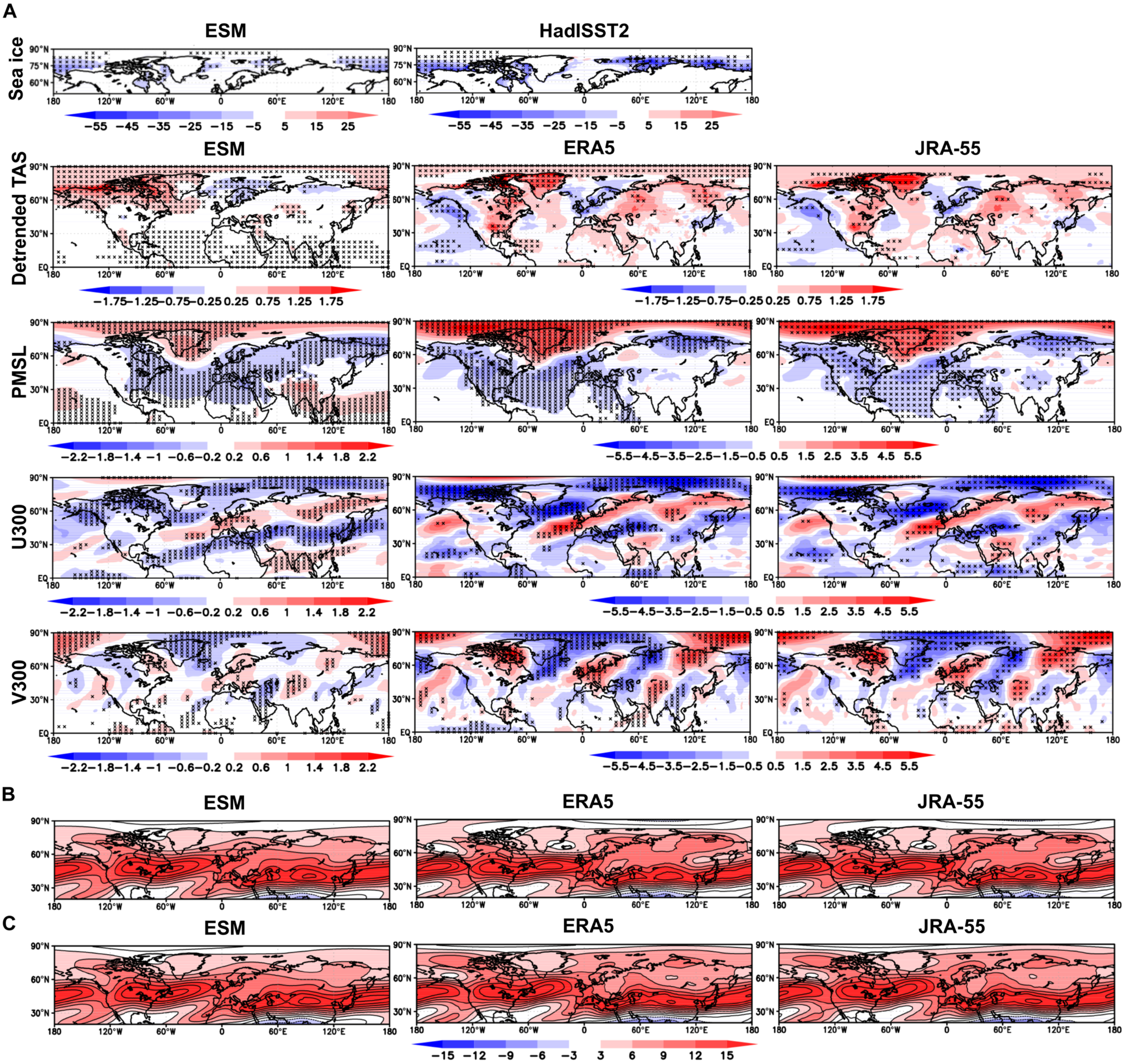

Fig. 4. Composite maps of climate variables related to ESAC SIC. (**A**) Differences in key climate variables as listed at the left for CMIP6 models and ERA5 and JRA-55 observations over the NH between the eight lowest ESAC SIC years and eight highest ESAC SIC years in JJA, 1979–2015. (**B**) Average absolute zonal wind (u) at 300 hPa (u300) for lowest extreme eight SIC years in JJA. (**C**) Average absolute u300 for highest extreme eight SIC years in JJA. The Met Office Hadley Centre sea ice and SST dataset, version 2.2 (HadISST.2) differences are also shown on the row. ESM denotes the ensemble mean difference from the 12 models. Black crosses represent statistical significance at the 5% level using a two-sided *t* test.

significant low-pressure anomalies are seen near the Arctic coast of Siberia in all three summer periods in both models and observations. Similar diagnostics were also created for the five most extreme pairs of years and are quite similar, so the results are not sensitive to a reasonable number of chosen extreme pairs of years.

Because of a general warming in near-surface air temperature (TAS) in models and observations, markedly greater for the models, we show differences in linearly detrended TAS. All the other variables shown in Fig. 4 and fig. S2 are not detrended. Precipitation is discussed later. The observed and modeled detrended TAS differences are also broadly similar, with an area of statistically significant warmer conditions in extreme low SIC years covering the ESAC region in the models. There is a rather weaker warm signal in this region in ERA5, but JRA-55 is closer to the models here in all three summer periods. Table S2 clarifies the SNAO results by showing the mean standardized modeled and observed SNAO regional indices corresponding to the difference

between eight lowest ESAC SIC cases and eight highest ESAC SIC cases for 1979–2015. The index is positive when PMSL is relatively higher over the North Sea node and negative when the PMSL is relatively higher over Greenland. The negative differences reflect the fact, as expected in all three summer periods, that low ESAC SIC relative to high SIC shows relatively low PMSL over the North Sea mode and high PMSL over the Greenland node. All three summer periods give significant negative differences at the 1% level in the models. In the observations, JJA and JA differences are significant at the 5% level, but in August with more intrinsic variability, the difference is not significant. The absolute differences in averages of the models are understandably less than for the observations because of ensemble averaging and the fact that underlying SNAO patterns vary quite a lot, though none are essentially different (fig. S3; see also Materials and Methods). Overall, table S2 confirms that it is very likely that ESAC SIC variations are linked to the variability of the SNAO. The PMSL maps (Fig. 4A and fig. S2) underline the much wider extent of this relatively strong connection as they show modeled and observed PMSL differences significant at the 5% level across the NH in all three summer periods.

We next examine differences in the zonal component of 300-hPa winds in Fig. 4A and fig. S2. There are again strong similarities between the modeled and observed differences, most of which are widely significant at the 5% level. A consistent feature in JJA, JA, and August in both models and observations is an increase in the westerly flow over the mid-latitude Atlantic from the central Atlantic in low relative to high ESAC SIC years. This feature extends across the UK and southern Scandinavia across the high latitudes of Eurasia, with often a sharp decrease in westerly flow to the north of this band. This is consistent with the well-known southward movement of the jet stream in a negative phase of the SNAO (*40*, *42*, *56*). Figure 4A and fig. S2 also show differences in the meridional v component of 300-hPa (v300) winds that reflect changes in the wave structure of 300-hPa winds. Strong similarities in wavelike anomalies are found between observations and models in all summer periods. They suggest an extensive Rossby wave response, clearest and strongest in the Greenland/North Atlantic/European sector but extending further downstream as far as China (*57*). Over the North Atlantic/European sector, the phases of the anomalous southerly and northerly v300 flow in the observations match those in the models well. This again suggests that the apparent changes in Rossby wave activity between the ESAC SIC extremes are indeed connected to ESAC fluctuations.

Last, we note that Fig. 4 (B and C) shows that the Arctic jet stream structure is quite similar in both extreme lowest and highest ESAC years in JJA; JA and August are similar. Thus, the background climatological Arctic jet stream climatology structure shown in Fig. 3 (K and L) for JJA and August remains almost the same for the full range of years with different ESAC SICs over the period of this analysis. The relatively small differences between Fig. 4B and Fig. 4C of course reflect the u300 differences in Fig. 4A. We now further test the hypothesis that SNAO variability could at least partly result from ESAC SIC forcing, rather than just the atmosphere forcing sea ice changes, using daily model and ERA5 data.

Is there a response from the SNAO following ESAC variations?

The results so far provide evidence for ESAC SIC fluctuations being connected to the SNAO variations. However, the distinction between forcing and response in coupled models and observations is hard to establish. Interactions between the ocean surface and the atmosphere can occur in both directions, the atmosphere affecting the ocean and the ocean the atmosphere. From a forecasting perspective, this interaction becomes a potential source of predictability if SIC variations lead variations in SNAO phases, as summer sea ice is skillfully predictable on seasonal timescales (*58*).

To investigate the relationship between sea ice and the SNAO, we use 10 of the CMIP6 models used in Fig. 4A for which daily data were available (Table 2, left). We used all 28 ensemble members with equal weight for each member (Table 2, left and see Materials and Methods). First, it is necessary to remove the very strong seasonal cycle from the daily SIC data especially to explore the lead-lag relationships between SIC and the SNAO, here its regional version. Second, daily data have a poor signal-to-noise ratio, so it is necessary to quite strongly average the daily data. Figure 5 shows a set of lead-lag correlations between SIC and the SNAO where the SNAO leads SIC on the left of the diagram and SIC leads the SNAO on the right. If no averaging is done, the rudiments of a signal can be seen on the daily timescale with a similar shape to Fig. 5 but with much less amplitude (not shown). The signal-to-noise ratio increases fairly rapidly with averaging time at first, then slowly with increased averaging time over the range 21 to 31 days. Figure 5 shows details for JJA and JA for the 31-day averaging time between ESAC SIC and the regional SNAO for the models, ERA5, and the average of JRA-55 and NCEP2. This gives marginally the highest correlations overall but a 25-day average is nearly as good. Figure 5 does depend on the fact that the SNAO can be identified as a similar pattern on short summertime scales down to the daily (*40*). Figure 5 (A to D) shows lead and lag correlations and their uncertainties for JJA and JA for the ERA5, the average of JRA-55 and NCEP2 (Fig. 5, B and E), and CMIP6 (Fig. 5, C and F). Leads and lags are shown from the SNAO leading by 30 days to SIC leading the SNAO by 30 days. The important results are for the differences between the high and low years. We have separately analyzed ERA5 and the combination of JRA-55 and NCEP2 as ERA5 shows somewhat different lead-lag relationships from the other reanalyses, these being similar. This difference of behavior can be seen in the top two rows of Fig. 5. Sea ice is treated differently between ERA5 and the other two reanalyses (see Materials and Methods), so this may explain this difference in behavior.

Note that when the lower 95% level of each correlation is above zero in Fig. 5, this shows that the correlation is significantly different from zero purely because of sampling, e.g., from sampling the 12 models having a total of 28 ensemble members. This is different from the 5% confidence level for a real lead-lag correlation between SIC and the SNAO which for a 31-day averaging for a lead-lag of 20 days in JJA is assessed to be 0.57. Such critical values of the lead-lag correlation coefficient were calculated using a Monte Carlo approach. Here, 100 groups of two 1000-year normally distributed random time series were generated. Thirty-one– (Fig. 5) and 15-day running averages were applied to the data before calculating the lead-lag correlation coefficient, the same procedure used in the lead-lag correlation analysis. The mean values of 100 groups of the 95 and 99% rank of the 1000-year lead-lag correlation coefficients are then selected as the critical values for 0.05 and 0.01 significance levels. As examples for JJA, the lead-lag correlation coefficients must exceed the high values of 0.71 (0.83) and 0.57 (0.71) for a lead-lag of 20 days to pass a 5% (1%) significant test for 31- and 15-day running averages, respectively. The needed correlations for JA and August would be larger, and for a lead-lag near zero, the needed correlations would be

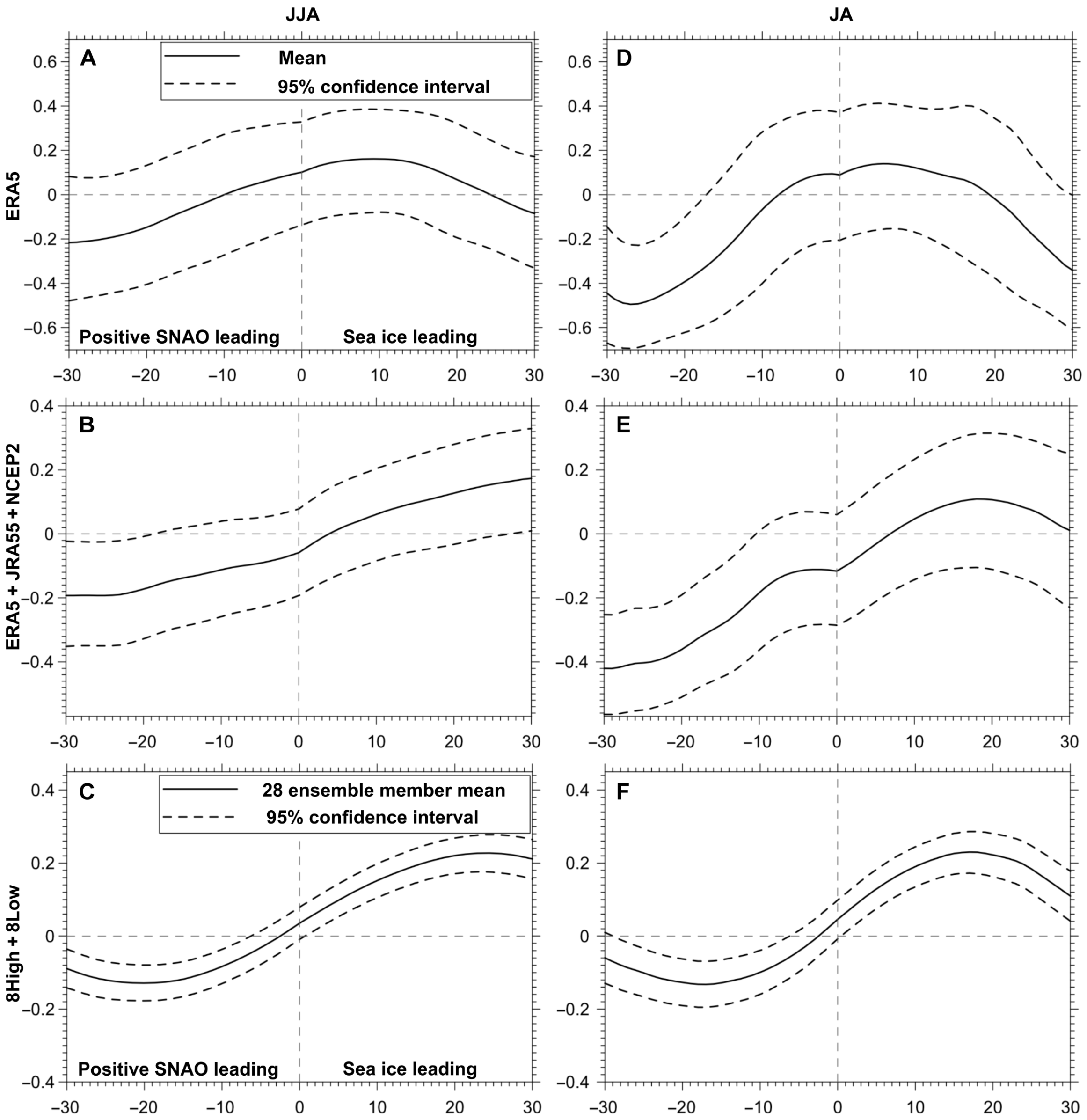

Fig. 5. Thirty-one–day running mean lead and lag correlations between the SNAO index and ESAC SIC in reanalysis and CMIP6 models for JJA and JA. (**A** and **D**) Difference between high SIC and low SIC in ERA5. (**B** and **E**) Difference in high minus low SIC in the combination of JRA-55 and NCEP 2. (**C** and **F**) Difference in high minus low SIC in the models. The *x*-axis scale is that of SIC lags and leads, shown lagging by up to 30 days (left) and leading by up to 30 days (right). The SNAO index is calculated on the basis of the regional difference [(55°W to 30°W, 60°N to 75°N) − (5°W to 15°E, 50°N to 60°N)]. The seasonal cycle of the daily time series has been removed.

slightly smaller. Thus, the time-averaged lead-lag analysis in Fig. 5 can indicate whether lead-lag relationships may exist, but there are too few data in a summer season to assess their true statistical significance. We call these appreciable correlations.

We first note that for differences between the eight highest ESAC SIC years and the eight lowest, the ERA5 observations (Fig. 5, A and D) only show appreciable correlations for an SNAO lead over SIC in JA maximizing for a lead near 25 to 30 days. Despite the rather different shape of the lead-lag correlations, the JRA-55 and NCEP 2 reanalyses show an appreciable lead of the SNAO compared to SIC at around 30 days and a marginally appreciable lead of SIC over the SNAO at a lead of about 30 days. By contrast, the CMIP6 data show much clearer if modest and very appreciable lead and lag correlations, noting that correlations for the CMIP6 analyses are based on about 30 times as much data as for the ERA5 analysis. First, we analyze the situation when SIC leads the SNAO on the right-hand side of Fig. 5. The largest positive CMIP6 correlations for SIC data leading the SNAO in both JJA and JA (Fig. 5, C and F). These correlations are both appreciable if modest at 0.23. They occur when positive (negative) SIC anomalies leads the positive (negative) SNAO by about 24 days (JJA) or about 17 days (JA). Broadly similar weaker but similarly shaped correlations are seen for the extreme high and low SIC sets of extreme years individually, clearest for JA though not always statistically significant in the

above sense. Figure S4 shows results for August where because of the reduced number of days in 1 month, only averaging up to 15 days can be carried out. Despite this, the differences in SIC extremes have quite similar maximum correlations to those in Fig. 5, the 15-day correlation reaching 0.20 for an SIC lead time of about 9 days and significantly different from zero. The high and low extreme years also show correlations marginally significantly different from zero for an SIC lead over the SNAO near 10 days. The reanalyses do not give any correlations significantly different from zero in August.

For the difference between the SIC extremes JJA and JA when the positive (negative) SNAO leads negative (positive) ESAC SIC, the models are maximally negatively correlated (JJA and JA = −0.13) when the SNAO leads by about 20 and 17 days, respectively. The model maximum negative correlation is also significantly different from zero in August for a lead time of about 10 days. For ERA5 as well as the combination of the other two reanalyses, the maximum negative correlation is just significantly different from zero in JJA but clearly significantly different from zero in JA at an SNAO lead time around 25 to 0 days. The model analyses for August in fig. S4 give slightly stronger results with a maximum negative correlation of −0.28 for an SNAO lead time of about 9 days. These results are at first glance difficult to understand because Fig. 3I shows that, as expected from an observed increase in PMSL over the SAEC region with a negative SNAO, downward surface net radiation is enhanced. However, the same change of sign occurs in some relationships between the winter NAO and SIC. Thus, daily lead-lag correlations between Barents-Kara SIC and the NAO also show a negative correlation when the NAO leads and a positive correlation when the ice leads (*59*) This makes physical sense because a positive NAO corresponds roughly to a northward jet shift resulting in the jet pointing more directly toward the Barents-Kara region. This then leads to associated changes to ice drift, wind stress, and the advection of additional warm air toward the polar region, all of which can reduce sea ice (*60*, *61*). A possibly related explanation is beyond the scope of this paper but may be related to transient ESAC atmospheric circulation tendencies before the development of a given phase of the SNAO. We conclude by noting that Fig. 5 provides evidence of a modest model influence of ESAC SIC on the SNAO with a lead time of several weeks and a weaker but just statistically significant influence, in the above sense, with a lead time of about 30 days in the JRA-55 and NCEP-2 reanalyses.

Can Polar Amplification Model Intercomparison Project experiments give further insights?

Another possible source of information about the links between SIC and the SNAO could come from the Polar Amplification Model Intercomparison Project (PAMIP) model experiments (*62*) However, the 100 members of 1-year (2000) simulations from the coupled ocean-atmosphere time slice experiments participated in PAMIP cannot well represent the many fluctuations in sea ice over the ESAC region that we investigate with CMIP6 and thus the responses of climate variables to the substantial variations in sea ice over this region. In addition, simulations from the atmosphere-only time slice experiments investigating regional forcing have only carried out experiments for two regions, the Sea of Okhotsk and the Barents-Kara Sea, that are not the focus of this work. Therefore, in this study we will not use PAMIP simulations.

Are other factors correlated with SNAO and ESAC SIC changes?

In some cases, apparent links between the winter NAO and sea ice may be explained by forcing from the tropics that could affect both sea ice and NAO (*34*), as shown for links between the tropics and high latitudes in summer (*55*). Figure S5 shows differences in precipitation globally between the eight lowest and eight highest ESAC SIC years in CMIP6 models and for ERA5 and JRA-55 in JJA, JA, and August. The wet negative SNAO southern node over UK extending to Scandinavia (*40*) is clear in the models in these summer periods, corresponding to reduced SIC in the ESAC region with more restricted but still statistically significant enhanced rainfall over this region in ERA5, though not in JR-55. In the models, this area of enhanced precipitation extends across the high Arctic Eurasian latitudes in all summer periods, corresponding to the enhanced jet stream in Fig. 4 (B and C) shown for JJA and where JA and August are similar. An appreciable drying over the eastern half of the ESAC region is clear and largely statistically significant in both ERA5 and the models, though hardly visible in JRA-55. ERA5 shows one other statistically significant precipitation region, a significant and consistent enhancement of precipitation in all summer periods in the narrow but long eastern North Pacific Intertropical Convergence Zone region. This may relate to the observed SNAO link with El Niño–Southern Oscillation (*40*). The models show this weakly in each summer period, but not significantly. Some partly statistically significant enhanced Sahel precipitation anomalies can also be seen in the CMIP6 models but not in ERA5.

Turning to SST, the SNAO appears to be affected by the summer Atlantic tripole SST pattern (*63*) such that a negative (positive) tripole (including warm (cool) SSTs in the tropical Atlantic) is associated with a negative (positive) SNAO index (*64*) and higher (lower) than normal Sahel rainfall as the Intertropical Convergence Zone moves anomalously north (stays anomalously south) (*65*). However, our particular subset of years based on extreme SIC in the ESAC region, though still linked to the SNAO, does not obviously reflect this relationship as viewed in the detrended TAS diagnostics in Fig. 4A, especially for ERA5. However, some evidence of a direct link exists between Sahel rainfall and the SNAO, independent of SST forcing (*66*). Furthermore, a direct if modest link of tropical West Pacific SSTs to the SNAO has been shown (*64*). This could arise mainly through tropical rainfall forcing as weakly suggested for the Tropical West Pacific by fig. S5, mainly in the models. This is faintly reminiscent of the winter NAO, which is much more strongly influenced by tropical rainfall forcing (*67*). However, Fig. 4 shows that differences for extreme ESAC SIC years in both observed and model TAS over the West Pacific show rather little evidence for an SST influence for our subset of years. A relatively stronger region of statistically significantly enhanced precipitation in the northern tropical west Pacific associated with low ESAC SIC relative to high SIC is seen in the models in all summer periods, slightly further south in ERA5. However, neither center is associated with obvious changes of TAS in Fig. 4A. We return to the topic of whether tropical SST may be an appreciable forcing factor for the JJA and JA SNAO in our main analysis period of 1979–2015 when discussing Rossby wave influences below.

What do AMIP models show?

Figure 5 provides appreciably, in the sense described above, statistically significant, though only fairly modest in size, evidence of a possible influence on the SNAO by ESAC SIC using coupled models.

This raises the question of whether we can use AMIP models to investigate how important observed variations in SIC in the ESAC region might be for directly forcing the SNAO or whether the coupling is so important that AMIP models do not produce realistic results.

Table 2 (right) shows 12 AMIP models chosen to compare with the CMIP6 models (*50*). The total AMIP ensemble size is 29. Figure 6 shows results laid out for JJA in the same way as in Fig. 4A where the eight lowest observed extreme SIC years in JJA in the ESAC region are compared to the eight highest years in CMIP6 models, ERA5 and JRA-55. The model sea ice years are now of course those observed. The detrended TAS AMIP results bear some relation to ERA5 and JRA-55 but are not as similar in some regions as the coupled CMIP6 patterns, especially over Europe though warming over the ESAC region is seen in AMIP as would be expected. The biggest differences come in the PMSL patterns. There is no sign of a negative SNAO, the sign, if anything, being reversed. Thus, the high PMSL anomaly over the Arctic is entirely absent and thus very different from the reanalyses. The anomalous jet streams reflected by u300 are also poorly related to the picture provided by the reanalyses over much of the NH. The AMIP model responses are very dissimilar at the surface as measured by PMSL in higher latitudes, though more similar in the subtopics and tropics. We conclude that there may be a response in the AMIP models at 300 hPa that bears some similarity in v300 to that of CMIP6, but the jet stream response is essentially absent. This suggests that in the AMIP models the strong changes of ESAC sea ice are only very weakly forcing the atmosphere. Given the widespread realistic coupled model responses in Fig. 4, especially the highly statistically significant PMSL responses, together with the modest statistically significant model results in Fig. 5, this lack of realistic AMIP atmospheric responses to extreme ESAC SIC variations indicates that coupling is probably essential for exploring Arctic SIC interactions with the summer NH atmosphere. However, a note of caution is that it is still possible that the coupled models are less responding to ESAC SIC changes than to internal atmospheric variability. Thus, if the ESAC-SNAO correlation in the coupled system is actually due to a separate atmospheric phenomenon forcing both ESAC ice and the SNAO, then the ESAC-SNAO correlation would vanish in our AMIP simulations, because this atmospheric phenomenon would be unable to affect the prescribed sea ice. For example, the quite frequent result that in winter the negative phase of the NAO is influenced by reduced sea ice in the Barents-Kara Sea has been strongly challenged as being mainly due to internal extratropical atmospheric

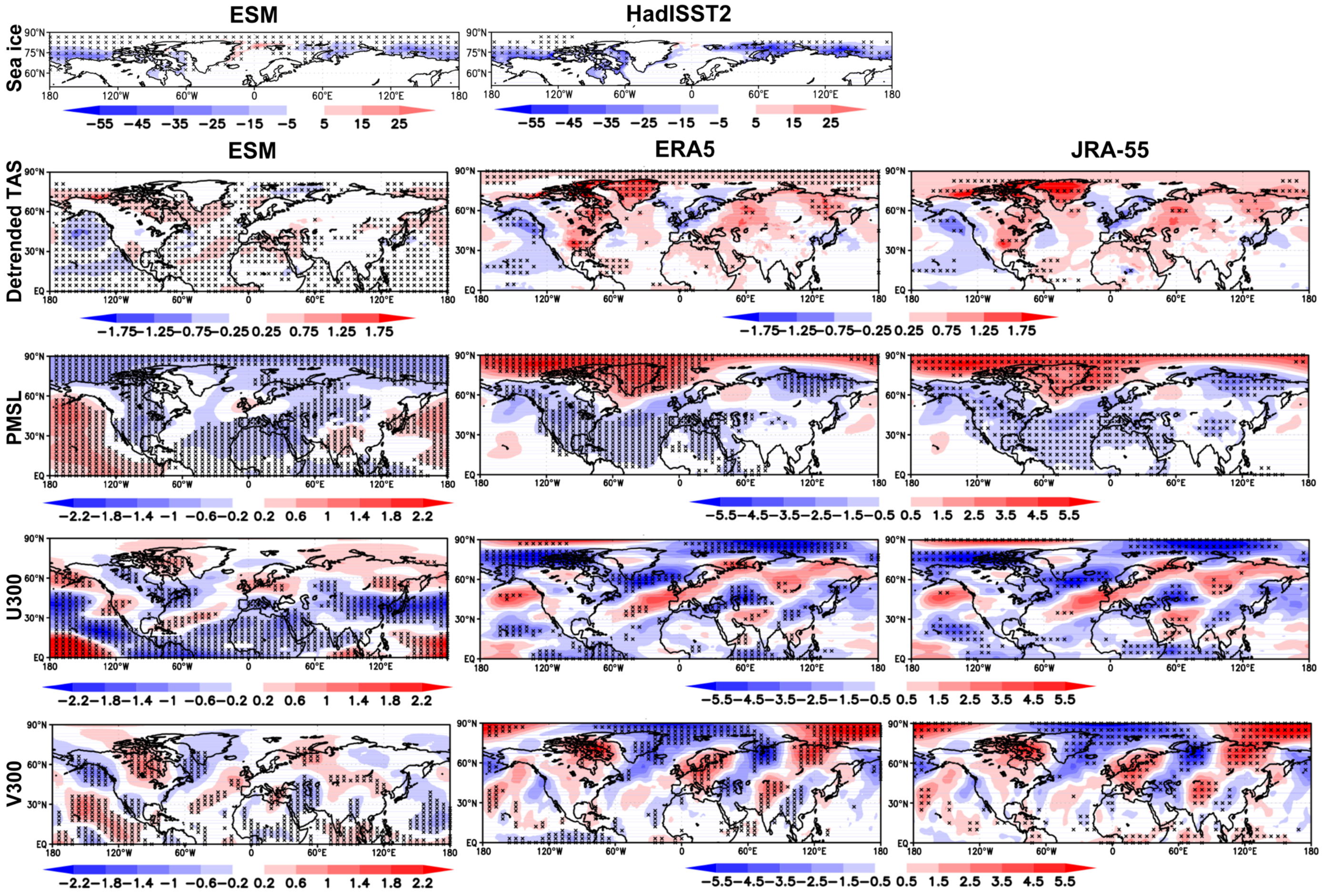

Fig. 6. Differences in key climate variables for JJA in AMIP models over the NH between the eight lowest ESAC SIC years and eight highest such ESAC years, 1979–2014. ESM denotes the ensemble mean difference from the 12 models and observations. The similar differences in the ERA5 and JRA-55 reanalyses. Black crosses represent significance at the 5% level using a two-sided *t* test.

variability (*68*) or a combination of this and tropical forcing (*34*). We investigate the latter further below.

Stream function differences between extreme SIC years

CMIP6 model and 300-hPa reanalyses in Fig. 4 suggest that an alternating pattern of positive and negative v300 winds downstream of the ESAC area is a prominent feature of the difference in atmospheric circulation at that level between very high and very low SIC ESAC sea ice summers. Here, we investigate this more closely by calculating 300-hPa stream function anomalies (*69*) for the same years. Stream function analyses do not overemphasize the extratropics, as do geopotential heights, for identifying features related to atmospheric Rossby waves (see Materials and Methods).

The stream function analysis (Fig. 7) at 300 hPa shows a series of observed positive and negative centers commencing in the ESAC region and propagating southeastward toward the east Asian tropics. Concentrating first on observations in JJA, there are five centers of alternate sign commencing with a center at high latitudes over Greenland extending westwards north of Alaska in both reanalyses. The five centers of alternate sign then extend southeast toward a negative center about 120°E over China. A second negative center exists over the North Atlantic and northwest Europe followed further southeast by a significant positive center over southeast Europe and western Asia followed by a further negative center over North India/Tibet, a positive center to its east, and lastly a negative center over eastern China. JA and August show a similar pattern that is overall slightly stronger still. This pattern and its southeastward tilt are consistent with a Rossby wave propagating from high latitudes toward the tropics and eastwards in the subtropical jet stream over Asia. Furthermore, the sequence of centers over the Greenland and east extratropical North Atlantic/ European region fits well with the surface SNAO pattern. This is consistent with SNAO being equivalent barotropic in character (*40*). However, there are further positive and negative centers over the higher latitudes of Asia and possibly the higher latitudes of the North Pacific, so the full response pattern is apparently more complicated than a single Rossby wave. Turning to the models, the stream function anomalies are broadly similar but weaker down to about 30°N, south of which the observations indicate the response largely dies out. There is also some evidence from the models that the stream function centers are a little stronger in JA and August. This is consistent with the finding that the SNAO is linked to the east Tibetan climate mainly in JA (*70*) and that the SNAO has a NH wide influence.

Could the tropics still directly affect the atmosphere in the ESAC region?

We further investigate the possibility that the tropics could affect the atmosphere in the ESAC region in summer. We first examine the sources and sinks of vorticity and hence Rossby waves (*69*, *71*) (see Materials and Methods) to see whether they relate to the features seen in Fig. 7 using ERA5. Locations of positive (left-hand two columns) or enhanced Rossby wave sources (right-hand column) at 300 hPa are shown in fig. S6 for JJA, JA, and August in red for the eight lowest SNAO index summers, the eight highest SNAO summers, and their differences. A key result is that the main Rossby wave source is over Greenland. Here in JA, it approaches 3×10^{-10} s^{-1} and remains there for both high- and low-SNAO extremes. There is evidence in the right-hand column that for the low SNAO relative to high, the Greenland Rossby wave source area extends westwards just north of the ESAC region, clearest in these diagrams in August. Referring back to the westerly wind diagnostics of Fig. 4 (B and C), this extension occurs on the poleward side of the weak u300hPa ESAC jet. However, there are no obvious signs of Rossby wave sources in any of the diagrams in the tropical West Pacific or

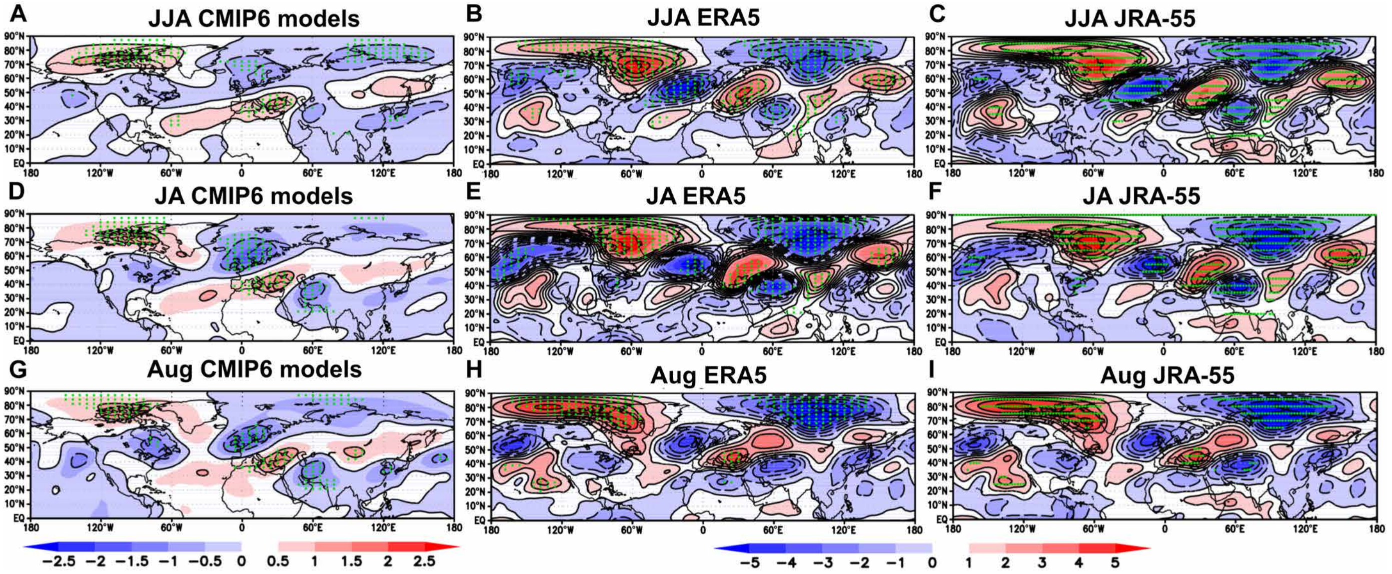

Fig. 7. Stream function differences at 300 hPa between the eight most extreme low SIC years and the eight most extreme high years, 1979–2015. (**A**) For JJA CMIP6 models. (**B**) For JJA ERA5 reanalysis. (**C**) For JJA JRA-55 reanalysis. (**D**) For JA CMIP6 models. (**E**) For JA ERA5 reanalysis. (**F**) For JA JRA-55 reanalysis. (**G**) For August CMIP6 models. (**H**) For August ERA5 reanalysis. (**I**) For August JRA-55 reanalysis. Stream function differences: red areas are positive; blue areas are negative. Note the change in scales for ERA5 and CMIP6, the increments of CMIP6 being 40% of those for ERA5 Thus, shade intervals are 0.4 10^6 m^2 s^{-1} (CMIP6) and 10^6 m^2 s^{-1} (ERA5 and JRA-55). Black crosses represent significance at the 5% level using a two-sided *t* test.

the tropics generally. Note also the several lower latitude Rossby wave sources associated with the NH-wide Rossby wave–like behavior in Figs. 4 and 7.

Figure S7 shows differences between the eight lowest SIC years and eight highest SIC years more clearly on a larger scale. In each of JJA, JA, and August, there is a relative Rossby wave source over the central and eastern part of the same areas just north of the ESAC region, again weakest in JJA and strongest in August in this way of showing the results. In each month, the area exceeding 5×10^{-11} s^{-1} can be regarded as a distinct Rossby wave source. In JJA, its maximum strength reaches about 8×10^{-11} s^{-1} comparable with one or two restricted areas in the higher latitudes. This value is weak though it corresponds to the apparent origin of the stream function centers in Fig. 7. In JA, the near ESAC source region is slightly stronger with a somewhat larger area above 5×10^{-11} s^{-1} exceeding 10^{-10} s^{-1} in a few local areas. In August, the near ESAC region shows a further slightly stronger signal, reaching 1.5×10^{-10} s^{-1} in a few local areas. Rossby wave sources preferentially occur where wind shear is high and where there is the divergence of its relative vorticity, and thus, its absolute vorticity can also be relatively high (Eq. 1). The ESAC region in the extreme SIC years (and climatologically) is clearly in the preferential latitude range for the upper tropospheric Arctic summer jet (*27*) as noted above (Figs. 3, K and L, and 4, B and C). However, the jet is relatively weak so the relative Rossby wave source difference is likely to be of moderate strength at most. We can compare these values with the climatological mean summer maximum Rossby wave source maxima between the Caribbean and Western Europe of about 10^{-10} s^{-1} or the maximum over the Pacific Ocean adjacent to northwest US of about 2×10^{-10} s^{-1} (*69*). Accordingly, fig. S7 lends additional support to the idea that stream function diagnostics in Fig. 7 are indeed related, in part at least, to atmospheric Rossby wave forcing from the ESAC region supported by an apparent modest increase in the signal in August relative to JJA when the differences in SIC are very liable to be largest. We note in addition that there are no obvious Rossby wave sources near or just north of the tropical West Pacific in figs. S6 and S7, or elsewhere in the tropics. Accordingly, from both Fig. 7 and fig. S5, we find no clear evidence of a direct forcing effect by tropical West Pacific rainfall on atmospheric circulation in the ESAC region which might influence SIC there.

We can extend our Rossby wave source analysis to consider all years over 1979–2015. First, fig. S8 shows time series in JA of the ESAC SIC, Rossby wave source strength, and the negative SNAO in standardized form. JJA is similar. The corresponding correlations between the negative sign of the SNAO, ESAC sea ice extents, and mean atmospheric Rossby wave source values at 300 hPa for the region 55°W to 150°W, 80°N to 90°N for both JJA and JA are shown in Table 3. All correlations are significant at the 5% level and are negative. Note that from fig. S6, the above region is actually a Rossby wave sink for high values of the SNAO, shown as a negative source. All correlations between Rossby wave source strength and ESAC SIC or the SNAO are in the range −0.37 to −0.47, only a little less in magnitude than those in Table 1 (top) between ESAC SIC and the SNAO though greater than in Fig. 5. Thus, the evidence is that the Rossby wave source over Greenland extends westwards over the ESAC region in our analysis period as ESAC SIC declines and the SNAO becomes more negative, suggesting a dynamical response of the atmosphere to ESAC SIC changes.

Last, we have further tested more directly whether SST in the tropics may affect our results by regressing the SNAO against worldwide SSTs down to 20°S in JJA against May SST and JA SNAO against June SST (fig. S9). This lead time by SST over the SNAO is necessary to unambiguously show evidence forcing of the SNAO by SSTs rather than include feedback of the SNAO on the SSTs. Figure S9 shows little evidence of statistically significant influences in our analysis period, especially one significant in both JJA and JA, though minor effects cannot be ruled out. In conclusion, we do not think that in our analysis period, 1979–2015, tropical SST influences are important for our ESAC SIC results.

DISCUSSION

The key question that the analysis in this paper is designed to answer concerns whether there is a link between the SNAO and the ESAC sea ice and whether there is any evidence for one forcing the other. For the period 1979–2015, we provide evidence of a link between the SNAO and Arctic Ocean SIC in the ESAC region. To achieve this, we use a combination of observations using three reanalyses, dynamical analyses using 12 CMIP6 coupled models and 12 AMIP models, all with realistic climatologies over the North Atlantic region. This analysis therefore uses a more comprehensive range of models than used before to study the interactions of summer Arctic sea ice on NH summer climate. We also investigate whether changes in Rossby wave forcing within the Arctic jet stream near the ESAC region might modulate the SNAO, resulting in a dynamical response of the atmosphere in that region.

We find consistent results between observational reanalyses and coupled CMIP6 models for links between the SNAO and Arctic sea ice, though the effects are modest. However, the coupled models have a strong SNAO response that gives a realistic set of teleconnections across the NH (Figs. 4 and 7). The key result is that, through an analysis of lead and lag relationships on multidaily timescales

Table 3. Correlations over 1979–2015 between the negative sign of the SNAO, ESAC sea ice extents and mean atmospheric Rossby wave source values at 300 hPa for the region 55°W to 150°W, 80°N to 90°N (negative values being sinks) for JJA and JA. This is the region shown as changing its Rossby wave source characteristics as ESAC SIC and the SNAO changes. All correlations are significant at the 5% level.

	JJA ESAC sea ice	JA ESAC sea ice	JJA SNAO	JA SNAO	JJA Rossby wave source	JA Rossby wave source
JJA Rossby wave source	−0.39	−0.39	−0.47	−0.50	1.00	
JA Rossby wave source	−0.45	−0.45	−0.37	−0.41	0.90	1.00

between ESAC SIC and the SNAO, we find in the CMIP6 models a lead of ESAC SIC influences on the SNAO on timescales in the region of 3 weeks (Fig. 5) with a maximum correlation of 0.23. This value is large enough for sampling problems to show it is significantly different from zero but not large enough to address the true level of significance of this result. There is also in the CMIP6 models a negative correlation significantly different from zero between the SNAO leading SIC on timescales nearer 4 weeks of a type that is seen in winter with the NAO in other regions of the Arctic (*59*). The reanalyses carried out in the same way only show a few marginal correlations different from zero where JRA-55 and NCEP 2 combined just show SIC leading the SNAO by about 30 days in JJA as different from zero and also negative (positive) SNAO leading high (low) SIC as different from zero. However, there are some differences between the lead-lag relationships ERA5 on the one hand and JRA-55 and NCEP 2 on the other. These may be related to the differences in the way SIC is handled (see Materials and Methods), The use of 12 CMIP6 coupled models and 31 ensemble members helps to much increase the model sample size compared to the that of the reanalyses.

In ERA5, we do find some statistically significant dynamical links with SIC through Rossby wave forcing, though the strength of the effects is modest (Table 3). However, the correlation between Rossby wave forcing and both the SNAO and SIC is statistically significant in both JJA and JA, and fig. S7 for JA shows how this occurs interannually. However, a potential disadvantage is that the models might also underestimate SIC forcing through insufficiently accurate climatological mean states (*13*), though the mostly reasonable individual model representations of the SNAO EOF in fig. S3 show that we have probably minimized this problem by a careful choice of model based on statistics of their climatologies over the North Atlantic region. There is also the potential problem, especially over the North Atlantic, of the climatic signal-to-noise paradox (*72*), which tends to lead to an underestimation of climate signals by coupled models unless the ensemble size is very large. Furthermore, the AMIP results do not support our results. However, a number of studies have indicated that in winter coupling between the ocean and the atmosphere is very important (*13*, *59*, *73*, *74*). Therefore, this is the most likely reason for the fact that the generally very good PMSL responses we see in the coupled models (Fig. 4) are missing in AMIP (Fig. 6). The complete absence of the negative SNAO signal in the AMIP results (Fig. 6), unexpectedly realistic in the coupled model results (Figs. 4 and 7), is, nevertheless unexpected as well because weak evidence of forcing by the differences in SIC in the AMIP experiments might be thought likely.

However, we note two further limitations of AMIP integrations. First, AMIP models, suffering from thermal damping (*75*), reduce the likelihood that interactions with the imposed SST will create conditions for strong enough atmospheric Rossby wave sources to develop. This is made all the more difficult by the high latitude of the ESAC area where, despite the existence of Arctic jets, the needed substantial gradients of relative vorticity for Rossby wave development to be favored tend to be modest. However, Arctic relative vorticity gradients in latitudes such as near the southern part of the ESAC region in the real world may well still be large enough sometimes to initiate Rossby waves, especially in summer and autumn (*76*). These may be enhanced by sufficient diabatic heating, e.g., from melting ice in the ESAC region.

Recent papers (*34*, *68*, *77*, *78*) have provided evidence that in winter Barents-Kara Sea interactions between SIC and the atmosphere are dominated by atmospheric forcing related at atmospheric internal atmospheric variability. Thus, there was no clear evidence of the SIC leading the atmosphere in lead-lag analyses in this region. However, an important point is that observational analyses of the strength of relationships between SIC and the atmosphere (e.g., correlation, regression or SVD analyses) can be misinterpreted as they will often reflect both atmospheric and SIC forcing. Thus, our suggestive though modest results in Fig. 5, with a maximum correlation of 0.23 with SIC leading the atmosphere over the ESAC region in JJA or JA by about 20 to 25 days for differences between low and high SIC, should be preferred as a truer measure of SIC forcing to observational analyses of the above type. Thus, correlation values exceeding 0.5 in Table 1 (top) between JJA and JA ESAC SIC and the SNAO are likely overestimates when taken as a measure of SIC forcing alone (*78*). Accordingly, as discussed in the section on AMIP results, some of the coupled model results we examine might still have a component related to extratropical internal atmospheric variability that remains to be uncovered.

A number of papers suggest that other factors like SST also may modulate the SNAO as described in Introduction, so SIC influences are only a partial potential explanation of forced SNAO variability. However, in most of our analysis period of 1979–2015, there is evidence that the influences of SST on the SNAO may be rather small (fig. S9), especially from the main potential influences, the North Atlantic tripole and the tropical West Pacific, and smaller than in the earlier decades (*55*). This is consistent with our Rossby wave source results, there being no obvious source in the North Atlantic or West Pacific (figs. S5 and S6). In addition, a consequence of the improved way we have defined the spatial extent of the SNAO shows that it explains appreciably more variance of summer PMSL over the North Atlantic region than our original definition (*40*). At about 40% for the whole summer, this is only a moderately less percentage of total variance than exhibited by the NAO in winter. However, some details of the southern node of the SNAO pattern are likely to have varied over time. This may be important for the influences of SST and thus overall predictability (*55*).

Our key result that ESAC SIC may influence the SNAO might also modestly contribute to seasonal predictability of the SNAO. Recently, a factor that has been found that influences the SNAO, and thus its year-to-year character and predictability, is the state of the Arctic stratospheric winds in spring before the summer stratospheric easterlies develop (*79*). This only contributes when these winds are strong enough but it is the first clear evidence of some predictability. Our results may contribute further to predictability if a forecasting model has a sufficiently good interaction between the SNAO and ESAC Arctic sea ice. Thus, routine seasonal forecasts are now made of Arctic sea ice (*58*) (and, e.g., www.metoffice.gov.uk/research/climate/seasonal-to-decadal/long-range/arctic-sea-ice as of 28 April 2024).

In addition, we provide evidence that the SNAO influence extends over much of Eurasia, adding further to the now well-known influences of the SNAO on Eurasian summer climate and bringing together the regional results shown in a number of papers. Not all the areas shown as statistically significant have been commented on, but the Mediterranean, China, and Tibet have been discussed in some detail. However, Fig. 7 shows that much of the NH north of 25°N is affected (*40*, *43*, *57*, *70*, *80*–*82*), especially Eurasia and parts of North America. Nevertheless, internal variability may

substantially limit SNAO predictability unless other causes of SNAO forcing can be found. Future research needs to be carried out with a larger coupled multimodel ensemble having good NH jet stream climatologies. Experimental designs should attempt to isolate influences of SIC variations, influences of worldwide SST, and any additional influences of tropical rainfall variations on the SNAO, including the Sahel (*83*), in addition to quantifying internal variability.

Last, our results may have relevance for climate change. Our high ESAC sea ice years are all in the period 1979–1996 and the low sea ice years all in the later period 2007–2015. This reflects recent decadal changes in the SNAO to a more negative phase overall. Can this hitherto unexplained behavior of the SNAO be understood, and will it relatively soon reverse strongly under climate warming (*40*)? Thus, climate change projections tend to show marked increases of summer anticyclonicity over northwest Europe (*84*, *85*).

MATERIALS AND METHODS

Observations

The paper uses both directly observed data and atmospheric reanalyses which integrate observed atmospheric data with weather forecasting models. The key observed dataset is the HadISST2.2.0.0 SIC dataset available from www.metoffice.gov.uk/hadobs/hadisst2/index.html (as of 8 December 2020).

This is an update from HadISST 2.1.0.0 (*48*). HadISST2.2.0.0 is a monthly globally complete SST and sea ice dataset starting in 1850 where the sea ice data have a resolution of 1° × 1° for both the Arctic and Antarctic. The SIC are based on a combination of satellite and in situ data. HadISST2.2.0.0 SST data are currently not publicly available. There are additional historical as well as more recent sea ice data in HadISST2.2.0.0 compared to HadISST2.1.0.0. These slightly affect SIC trends in the Arctic in the period of our analysis. HadISST2.2.0.0 is updated regularly.

We use the Twentieth Century Reanalysis dataset, version 3 (20Cv3) (*86*) to provide PMSL data (https://psl.noaa.gov/data/gridded/data.20thC_ReanV3.html as of 20 January 2021) for calculating the various versions of the SNAO. However, these data are available only from 1836 to 2015, so we update this using the National Centres for Environmental prediction Reanalysis (NCEP) R1 PMSL analysis (*87*) that commences in 1948 (https://psl.noaa.gov/data/gridded/data.ncep.reanalysis.html as of 22 January 2021), slightly adjusted to be consistent with 20Cv3. This is updated monthly. Only monthly PMSL data are used for the SNAO in this paper. For the maximum covariance analysis between North Atlantic/European PMSL and Arctic SIC variations over 1979–2015 (see the "Statistical methods" section below), we use the ERA5 Reanalysis (*49*) accessible from https://cds.climate.copernicus.eu/cdsapp#!/dataset/reanalysis-era5-single-levels-monthly-means?tab=form as of 21 January 2021. We accessed this dataset via KNMI Climate Explorer (https://climexp.knmi.nl/). For maximum covariance analysis, the ERA5 PMSL data are used at reduced 3° × 3° resolution because of the computational demands, and for the Rossby wave source analyses, we used ERA5 at 0.75° × 0.75° resolution to reduce noise in the diagnostics. We also used the JRA-55 (https://search.diasjp.net/en/dataset/JRA55 as of 7 March 2024) and NCEP-DOE AMIP-II (R-2) (https://psl.noaa.gov/data/gridded/data.ncep.reanalysis2.html as of 22 March 2024) reanalyses for some of the results. A complication exists for interpreting the observed averaged lead-lag analyses of SIC and the SNAO in Fig. 5. The external datasets used for SIC are different among ERA5, JRA-55, and NCEP-2. The Ocean and Sea Ice Satellite Application Facilities (OSI SAF) SIC from the European Organisation for the Exploitation of Meteorological Satellites is used in ERA5. Until August 2007, this is the SI SAF (409a) version and from September 2007 onward: the OSI SAF (operational) (*49*). Daily data on SIC conditions in JRA-55 are obtained from COBE-SST (Centennial In Situ Observation-based Estimates of the Variability of Sea Surface Temperatures and Marine Meteorological Variables) (*53*). The SIC in NCEP-2 follows the sea ice specifications in AMIP II (*54*). In addition, the SIC in ERA5 is fractional, while both JRA-55 and NCEP-2 have a binary representation with which the sea is completely ice covered when SIC is above 55% (50% for NCEP-2). Sea ice thickness is set to 1.5 m in ERA5 and 2 m for both JRA-55 and NCEP-2 reanalyses. JRA-55 and NCEP-2 have a small conductive heat flux value unlike ERA5. A general warm bias seen in ERA5 is reduced in both JRA-55 and NCEP-2. However, a cold bias is produced in the boundary regions in both JRA-55 and NCEP-2, such as the Barents-Kara Sea, Laptev Sea, and Chukchi Sea (*88*).

We used HadISST 2.2.0.0 Arctic SIC at a resolution of 2° × 2° degrees. For comparisons between CMIP6 models and observations, we use ERA5 at its full resolution of 0.25° × 0.25°, taken as above. We use ERA5 for u and v components of wind at 300 hPa, PMSL, precipitation, and TAS. We also tested diagnostics of u200hPa and u500hPa related to ESAC SIC to lastly choose 300 hPa as the most responsive level to use for the wind and dynamical diagnostics described here.

Climate models

The data used come from the CMIP6 historical (1979–2014) and shared socioeconomic pathway 2-4.5 (SSP 2-4.5) scenario (2015) runs (*50*) and include anthropogenic and natural forcings. Thirty-seven CMIP6 models were initially chosen (downloaded from https://esgf-data.dkrz.de/search/cmip6-dkrz/ accessed 23 August 2021). These were reduced to the 12 models having the most skillful climatology over the North Atlantic region using the diagnostics in table S3. Thus, the original set of models listed in table S3 is ranked according to their skill in reproducing the observed climate of PMSL over the extratropical North Atlantic and Europe (25°N to 90°N, 80°W to 40°E). Testing was done using ERA5 with a maximum resolution of 0.75° × 0.75° during the climatological 30-year period 1981–2010 in JJA and JA. The year 1979 was the first model year chosen for the analyses because this was the year when ERA5 data were originally first available, though they are now available back to 1940. However, another reason is that HadISST2.2.0.0 SIC is generally most reliable from 1979 because of the availability of comprehensive good quality satellite data (*48*). The two measures of model skill are correlated at the resolution of 0.75° × 0.75° as the ERA-Interim and root mean squared error. The chosen 12 models are listed in Table 2 (left) on the basis of a ranking of these statistics giving 31 ensemble members. As a result, key model results are all based on the differences between 248 extremely high and 248 extremely low SIC cases. However, only 10 models have daily data for the analysis in Fig. 5, so this used to create 28 daily ensemble members (Table 2, left). Ensemble members have equal weight so that different models have different weights. In Fig. 5, we calculate mean lead and lag correlations for the SNAO and ESAC SIC and their 95% confidence levels for (i) all data over 1979–2015 for JJA, (ii) the eight lowest concentration sea ice years, and (iii) the eight highest concentration sea ice years. Here, the regional SNAO index is convenient

for analyzing daily CMIP6 data, overcoming the problem that different climate models may have differing SNAO EOF patterns. Though this may affect the representativeness of the regional SNAO in some models, this is not a serious problem as shown by the patterns of the first JJA model EOFs in fig. S3. All the patterns resemble the observed JJA EOF of Fig. 1 to some extent, several models being very similar. The percentages of variance explained, here are nominally for 1981–2010 and vary somewhat, though none seem unrealistic. The overall average is close to that explained by the JJA SNAO EOF in Fig. 1. Similar results are found for JA and August (not shown). As a result, the likelihood of identifying a reasonably realistic SNAO index using this set of 12 models is well supported by fig. S3.

Besides the CMIP6 historical runs, AMIP simulations, in which the SSTs, SICs, and external forcings (e.g., CO2 concentration) are prescribed on the basis of observations (*50*), were also used to assess the possible response of atmospheric and land components to prescribed SSTs and SICs. The 12 most skillful models were chosen from the 36 candidate models in a similar way to the process of choosing the CMIP6 historical runs (table S4). The AMIP simulations cover the period from January 1979 to December 2014.

Dynamical methods

We use dynamical diagnostics of the sources of atmospheric Rossby wave and their identification through the use of stream functions in Fig. 7 to investigate possible mechanisms of the influence of ESAC SIC variations on the atmosphere. In our case, dynamical diagnostics of 300-hPa height over the ESAC region was of most interest, being of most variability. The strength of a Rossby wave source or sink in a region of the atmosphere at a given level is related to the propagation of Rossby waves (*71*)

$$\partial\zeta / \partial t + \mathbf{v}_{\psi} \cdot \nabla \zeta = -\nabla \cdot (\mathbf{v}_{\chi} \zeta) \tag{1}$$

Here, ζ is the absolute vorticity, $\mathbf{v}_{\psi}$ is the nondivergent or rotational wind vector, where its components u_{ψ} and v_{ψ} here are confined to a horizonal level and $\mathbf{v}_{\chi}$ is the corresponding divergent component of the wind.

Here, we diagnose differences in 300-hPa Rossby wave source globally in Fig. 7 between the 8 years over 1979–2015 with extremely low SIC and the 8 with extremely high SIC in the ESAC region using the westerly, u, and southerly, v, components of the wind at 300 hPa. This level is almost always in the upper troposphere at high latitudes, unlike 200 hPa, and shows a stronger Rossby wave source. Equation 1 shows that the stream function ψ is important as it measures the nondivergent component of the wind which, as the left-hand side of Eq. 1 shows, is the component that propagates Rossby waves. The stream function can be calculated from the individual wind components using the vector relation

$$\mathbf{v}_{\psi} = \mathrm{k} \times \nabla\psi \tag{2}$$

In Fig. 7, the zonal mean stream function has been removed because zonal mean summer winds are mostly westerly at 300 hPa in the extratropics, and much larger than the v component, rendering this necessary to highlight any quasi-zonal wave-like responses.

Statistical methods

We first use EOF analysis to extract the pattern for identifying the SNAO in JJA, JA, and August separately. This is used to extract orthogonal spatial patterns or EOFs of a vector $\mathbf{x}$ of p variables explaining the most variance. Here, we look for the linear vector function $\mathbf{a_1}^{\mathrm{T}}\mathbf{x}$ of the elements of $\mathbf{x}$ with maximum variance where T is the transpose

$$\mathbf{a_1}^{\mathrm{T}}\mathbf{x} = a_{11}x_1 + a_{11}x_2 + a_{12}x_3 \ldots\ldots + a_{1p}x_p = \sum_{j=1}^{p} a_{1j}\, x_j \tag{3}$$

In Eq. 3, the p a_{1j} give the first EOF pattern. Next, we look for a linear function $\mathbf{a_2}^{\mathrm{T}}\mathbf{x}$ uncorrelated with $\mathbf{a_1}^{\mathrm{T}}\mathbf{x}$ that maximizes the remaining variance to give the second pattern, etc. In our case, the SNAO pattern of weights a_{1j} is much the most important or very clearly the first EOF. Remaining EOFs of course are orthogonal to the SNAO and thus have independent time series for the same period that the spatial patterns are calculated. A basic summary (*71*) and a detailed explanation of EOF analysis is readily available in many publications (*89*). It is possible to estimate the statistical significance of an EOF pattern, but we have not done this here for the SNAO as this is a well-accepted pattern.

We also use a multivariate method known as maximum covariance analysis (*89*), sometimes referred to as SVD, which identifies the covariance structure between two different datasets. Climate applications of this method have been described in detail (*90*, *91*). We use this to compare the maximally covarying patterns of Arctic-wide SIC with PMSL in the North Atlantic/European region in JJA, JA, and August separately. The method extracts the pair of patterns and their associated time series from two sets of data that maximally covary with each other as measured by their covariance squared. As with EOF analysis, further patterns can be extracted from the residual variability remaining after the variability associated with the first set of patterns is subtracted from the datasets. We do not need to compute any of the lower variance patterns here. Each set of pattern loadings can be projected onto the original data to give a time series of the amplitude of the set of patterns. If the first calculated pattern (the left vector) is projected onto the left set of original maps (thus the same variable), the resulting time series is called homogeneous. If the first calculated pattern is projected onto the second or right original time series (the other variable), the resulting time series is called heterogeneous. Here, we only calculate homogeneous time series of the first set of left and right vectors in the form of Arctic SIC changes (the left vector) and the PMSL in the North Atlantic/European region (the right vector) as these turn out to be the dominantly covarying ESAC SIC and SNAO PMSL patterns. This method can be viewed as a form of principal component analyses carried out on the SIC and PMSL datasets so as to match up their time series as closely as possible (*89*).

For calculating the uncertainties in the running lead-lag correlations, we have adopted the following procedure. A Fisher z-transformation has been applied to the correlation coefficient (r) of each ensemble member of the CMIP6 simulations (or each year for the reanalysis) before calculating the 95% confidence level. The Fisher z-transformation of r is

$$z = \frac{1}{2}\ln\left(\frac{1+r}{1-r}\right) \tag{4}$$

Equations 5 and 6 below are then used to get the z values for the upper and lower confidence limits for a given lead/lag day from all ensemble members at the 95% confidence level

$$z_{\text{Lower limit}} = z_{\text{mean}} - z_{.95} * \mathrm{SD} / \sqrt{N} \tag{5}$$

$$z_{\text{Upper limit}} = z_{\text{mean}} + z_{.95} * \text{SD} / \sqrt{N} \quad (6)$$

where z_{mean} is the mean of z value for all members for the given lead/lag day; SD is their standard deviation; N is the number of members; $z_{.95}$ is the 95% confidence interval equal to 1.96. Then z_{mean}, $z_{\text{Lower limit}}$, and $z_{\text{Upper limit}}$ are converted to the mean, lower, and upper limits at the 95% confidence interval of correlation coefficient for all the members using Eq. 4

$$r = \frac{\exp(2z) - 1}{\exp(2z) + 1} \quad (7)$$

Last, when comparing the temporal correlation between two series, we noted whether the first few serial correlation coefficients were significantly different from zero in both series. If they differ from zero in only one series, the number of degrees of correlation freedom is not reduced. Thus, if N is the number of independent pairs of data for calculating the significance of a correlation coefficient, then the reduced effective number N_e when both datasets m and n are serially correlated is (*92*)

$$N_e = N \left\{ \sum_{\rho=-N+1}^{N-1} \left(\frac{1-|\tau|}{N} \right) [\rho_{mm}(\tau)\rho_{nn}(\tau)] \right\}^{-1} \quad (8)$$

where the summation is done over all serial correlations $|\tau| < N$ until they are statistically insignificant. However, if either all serial correlations ρ_{mm} or all ρ_{nn} are insignificant (effectively zero), then it can be seen that $N_e = N$. Because the first serial correlation coefficients of all versions of the SNAO, the SVD1 patterns and the Rossby wave source index used here over 1979–2015 or 1961–2019 are not significantly different from zero over 1961–2019, we can use their nominal $N - 2$ or 35 correlation degrees of freedom to estimate the significance of correlations with SIC and Rossby wave source averages, despite appreciable serial correlation in the ESAC SIC data.

Supplementary Materials

This PDF file includes:

Figs. S1 to S9

Tables S1 to S4

REFERENCES AND NOTES

1. J. A. Screen, I. Simmonds, The central role of diminishing sea ice in recent Arctic temperature amplification. *Nature* **464**, 1334–1337 (2010).
2. P. Chylek, C. K. Folland, J. D. Klett, M. Wang, N. Hengartner, G. Lesins, M. K. Dubey, Annual mean Arctic amplification 1970–2020: Observed and simulated by CMIP6 climate models. *Geophys. Res. Lett.* **49**, e2022GL099371 (2022).
3. J. C. Comiso, C. L. Parkinson, R. Gersten, L. Stock, Accelerated decline in the Arctic sea ice cover. *Geophys. Res. Lett.* **35**, L01703 (2008).
4. J. C. Comiso, Large decadal decline of the Arctic multiyear ice cover. *J. Climate* **25**, 1176–1193 (2012).
5. J. C. Stroeve, M. C. Serreze, M. M. Holland, J. E. Kay, J. Maslanik, A. P. Barrett, The Arctic's rapidly shrinking sea ice cover: A research synthesis. *Clim. Change* **110**, 1005–1027 (2012).
6. D. Budikova, Role of Arctic sea ice in global atmospheric circulation: A review. *Glob. Plan. Change* **68**, 149–163 (2009).
7. J. A. Francis, W. Chan, D. J. Leathers, J. R. Miller, D. E. Veron, Winter Northern Hemisphere weather patterns remember summer Arctic sea-ice extent. *Geophys. Res. Lett.* **36**, L07503 (2009).
8. J. E. Overland, M. Wang, Large-scale atmospheric circulation changes are associated with the recent loss of Arctic sea ice. *Tellus A* **62**, 1–9 (2022).
9. J.-S. Kug, J.-H. Jeong, Y.-S. Jang, B.-M. Kim, C. K. Folland, S.-K. Min, S.-W. Son, Two distinct influences of Arctic warming on cold winters over North America and East Asia. *Nat. Geosci.* **8**, 759–762 (2015).
10. C. Deser, R. A. Thomas, S. Peng, The transient atmospheric circulation response to North Atlantic SST and sea ice anomalies. *J. Climate* **20**, 4751–4767 (2007).
11. C. Strong, G. Magnusdottir, H. Stern, Observed feedback between winter sea ice and the North Atlantic oscillation. *J. Climate* **22**, 6021–6032 (2009).
12. V. A. Semenov, M. Latif, Nonlinear winter atmospheric circulation response to Arctic sea ice concentration anomalies for different periods during 1966–2012. *Environ. Res. Lett.* **10**, 054020 (2015).
13. D. M. Smith, N. J. Dunstone, A. A. Scaife, E. K. Fielder, D. Copsey, S. C. Hardiman, Atmospheric response to Arctic and Antarctic sea ice: The importance of ocean–atmosphere coupling and the background state. *J. Climate* **30**, 4547–4565 (2017).
14. J. A. Screen, C. Deser, D. M. Smith, X. Zhang, R. Blackport, P. J. Kushner, T. Oudar, K. E. McCusker, L. Sun, Consistency and discrepancy in the atmospheric response to Arctic sea-ice loss across climate models. *Nat. Geosci.* **11**, 155–163 (2018).
15. N. G. Kvamstø, P. Skeie, D. B. Stephenson, Impact of Labrador sea-ice extent on the North Atlantic Oscillation. *Int. J. Climatol.* **24**, 603–612 (2004).
16. J. Liu, J. A. Curry, H. Wang, R. M. Horton, Impact of declining Arctic sea ice on winter snowfall. *Proc. Natl. Acad. Sci. U.S.A.* **109**, 4074–4079 (2012).
17. F. Li, H. Wang, Relationship between Bering Sea ice cover and East Asian winter monsoon year-to-year variations. *Adv. Atmos. Sci.* **30**, 48–56 (2013).
18. B.-M. Kim, S.-W. Son, S.-K. Min, J.-H. Jeong, S.-J. Kim, X. Zhang, T. Shim, J.-H. Yoon, Weakening of the stratospheric polar vortex by Arctic sea-ice loss. *Nat. Commun.* **5**, 4646 (2014).
19. C. Deser, R. Tomas, M. Alexander, D. Lawrence, The seasonal atmospheric response to projected Arctic sea ice loss in the late twenty-first century. *J. Climate* **23**, 333–351 (2010).
20. D. M. Smith, R. Eade, M. B. Andrews, H. Ayres, A. Clark, S. Chripko, C. Deser, N. J. Dunstone, J. García-Serrano, G. Gastineau, L. S. Graff, S. C. Hardiman, B. He, L. Hermanson, T. Jung, J. Knight, X. Levine, G. Magnusdottir, E. Manzini, D. Matei, M. Mori, R. Msadek, P. Ortega, Y. Peings, A. A. Scaife, J. A. Screen, M. Seabrook, T. Semmler, M. Sigmond, J. Streffing, L. Sun, A. Walsh, Robust but weak winter atmospheric circulation response to future Arctic sea ice loss. *Nat. Commun.* **13**, 727 (2022).
21. M. Ogi, J. M. Wallace, Summer minimum Arctic sea ice extent and the associated summer atmospheric circulation. *Geophys. Res. Lett.* **34**, L12705 (2007).
22. Q. H. Ding, A. Schweiger, M. L'Heureux, D. S. Battisti, S. Po-Chedley, N. C. Johnson, E. Blanchard-Wrigglesworth, K. Harnos, Q. Zhang, R. Eastman, E. J. Steig, Influence of high-latitude atmospheric circulation changes on summertime Arctic sea ice. *Nat. Clim. Change* **7**, 289–295 (2017).
23. R. E. Petrie, L. C. Shaffrey, R. T. Sutton, Atmospheric response in summer linked to recent Arctic sea ice loss. *Q. J. R. Meterol. Soc.* **141**, 2070–2076 (2015).
24. M. A. Balmaseda, L. Ferranti, F. Molteni, T. N. Palmer, Impact of 2007 and 2008 Arctic ice anomalies on the atmospheric circulation: Implications for long-range predictions. *Q. J. R. Meteorol. Soc.* **136**, 1655–1664 (2010).
25. J. A. Screen, Influence of Arctic sea ice on European summer precipitation. *Environ. Res. Lett.* **8**, 044015 (2013).
26. R. E. Petrie, L. C. Shaffrey, R. T. Sutton, Atmospheric impact of Arctic sea ice loss in a coupled ocean–atmosphere simulation. *J. Climate* **28**, 9606–9622 (2015).
27. D. Coumou, G. Di Capua, S. Vavrus, L. Wang, S. Wang, The influence of Arctic amplification on mid-latitude summer circulation. *Nat. Commun.* **9**, 2959 (2018).
28. J. A. Screen, I. Simmonds, C. Deser, R. Tomas, The atmospheric response to three decades of observed Arctic sea ice loss. *J. Climate* **26**, 1230–1248 (2013).
29. G. Kay, N. Dunstone, D. Smith, T. Dunbar, R. Eade, A. Scaife, Current likelihood and dynamics of hot summers in the UK. *Environ. Res. Lett.* **15**, 094099 (2020).
30. B. Wu, R. Zhang, R. D'Arrigo, J. Su, On the relationship between winter sea ice and summer atmospheric circulation over Eurasia. *J. Climate* **26**, 5523–5536 (2013).
31. D. Guo, Y. Gao, I. Bethke, D. Gong, O. M. Johannessen, H. Wang, Mechanism on how the spring Arctic sea ice impacts the East Asian summer monsoon. *Theor. Appl. Climatol.* **115**, 107–119 (2014).
32. S. Lee, T. Gong, N. Johnson, S. B. Feldstein, D. Pollard, On the possible link between tropical convection and the Northern Hemisphere Arctic surface air temperature change between 1958 and 2001. *J. Climate* **24**, 4350–4367 (2011).
33. S. Lee, Testing of the tropically excited Arctic warming mechanism (TEAM) with traditional El Niño and La Niña. *J. Climate* **25**, 4015–4022 (2012).
34. J. L. Warner, J. A. Screen, A. A. Scaife, Links between Barents-Kara sea ice and the extratropical atmospheric circulation explained by internal variability and tropical forcing. *Geophys. Res. Lett.* **47**, e2019GL085679 (2020).
35. K. Deng, X. Jiang, C. Hu, D. Chen, More frequent summer heat waves in southwestern China linked to the recent declining of Arctic sea ice. *Environ. Res. Lett.* **15**, 074011 (2020).
36. R. Zhang, C. Sun, J. Zhu, R. Zhang, W. Li, Increased European heat waves in recent decades in response to shrinking Arctic sea ice and Eurasian snow cover. *npj Clim. Atmos. Sci.* **3**, 7 (2020).
37. B. Wu, Z. Li, Possible impacts of anomalous Arctic sea ice melting on summer atmosphere. *Int. J. Climatol.* **42**, 1818–1827 (2022).

38. Z. Wang, J. Walsh, S. Szymborski, M. Peng, Rapid Arctic sea ice loss on the synoptic time scale and related atmospheric circulation anomalies. *J. Climate* **33**, 1597–1617 (2020).
39. J. A. Screen, I. Simmonds, K. Keay, Dramatic interannual changes of perennial Arctic sea ice linked to abnormal summer storm activity. *J. Geophys. Res.* **116**, D15105 (2011).
40. C. K. Folland, J. Knight, H. W. Linderholm, D. Fereday, S. Ineson, J. W. Hurrell, The summer North Atlantic oscillation: Past, present, and future. *J. Climate* **22**, 1082–1103 (2009).
41. A. G. Barnston, R. E. Livezey, Classification, seasonality and persistence of low-frequency atmospheric circulation patterns. *Mon. Weather Rev.* **115**, 1083–1126 (1987).
42. B. Dong, R. T. Sutton, T. Woollings, K. Hodges, Variability of the North Atlantic summer storm track: Mechanisms and impacts on European climate. *Environ. Res. Lett.* **8**, 1–9 (2013).
43. H. W. Linderholm, C. K. Folland, A. Walther, A multicentury perspective on the summer North Atlantic Oscillation (SNAO) and drought in the eastern Atlantic region. *J. Quat. Sci.* **24**, 415–425 (2009).
44. E. Hanna, T. E. Cropper, P. D. Jones, A. A. Scaife, R. Allan, Recent seasonal asymmetric changes in the NAO (a marked summer decline and increased winter variability) and associated changes in the AO and Greenland blocking index. *Int. J. Climatol.* **35**, 2540–2554 (2015).
45. R. Allan, C. K. Folland, Atmospheric circulation. 1. Mean sea level pressure and related modes of variability. *State of the Climate 2017*. *Bull. Amer. Meteor. Soc.* **99**, Si–S310 (2018). http://dx.doi.org/10.1175/2018BAMSStateoftheClimate.1.
46. O. Ding, B. Wang, J. M. Wallace, G. Branstator, Tropical–extratropical teleconnections in boreal summer: Observed interannual variability. *J. Climate* **24**, 1878–1896 (2011).
47. A. Osso, R. Sutton, L. Shaffrey, B. Dong, Observational evidence of European summer weather patterns predictable from spring. *Proc. Natl. Acad. Sci. U.S.A.* **115**, 59–63 (2018).
48. H. A. Titchner, N. A. Rayner, The Met Office Hadley Centre sea ice and sea surface temperature data set, version 2: 1. Sea ice concentrations. *J. Geophys. Res. Atmos.* **119**, 2864–2889 (2014).
49. H. Hersbach, B. Bell, P. Berrisford, S. Hirahara, A. Horányi, J. Muñoz-Sabater, J. Nicolas, C. Peubey, R. Radu, D. Schepers, A. Simmons, C. Soci, S. Abdalla, X. Abellan, G. Balsamo, P. Bechtold, G. Biavati, J. Bidlot, M. Bonavita, G. De Chiara, P. Dahlgren, D. Dee, M. Diamantakis, R. Dragani, J. Flemming, R. Forbes, M. Fuentes, A. Geer, L. Haimberger, S. Healy, R. J. Hogan, E. Hólm, M. Janisková, S. Keeley, P. Laloyaux, P. Lopez, C. Lupu, G. Radnoti, P. de Rosnay, I. Rozum, F. Vamborg, S. Villaume, J.-N. Thépaut, The ERA5 global reanalysis. *Q. J. R. Meterol. Soc.* **146**, 1999–2049 (2020).
50. V. Eyring, S. Bony, G. A. Meehl, C. A. Senior, B. Stevens, R. J. Stouffer, K. E. Taylor, Overview of the Coupled Model Intercomparison Project Phase 6 (CMIP6) experimental design and organization. *Geosci. Model Dev.* **9**, 1937–1958 (2016).
51. R. E. Livezey, W. Y. Chen, Statistical field significance and its determination by Monte Carlo techniques. *Mon. Weather Rev.* **111**, 46–59 (1983).
52. R. W. Kolstad, J. A. Screen, Nonstationary relationship between autumn Arctic sea ice and the winter North Atlantic Oscillation. *Geophys. Res. Lett.* **46**, 7583–7591 (2019).
53. S. Kobayashi, Y. Ota, Y. Harada, A. Ebita, M. Moriya, H. Onoda, K. Onogi, H. Kamahori, C. Kobayashi, H. Endo, K. Miyaoka, K. Takahashi, The JRA-55 reanalysis: Generale specifications and basic characteristics. *J. Meteorol. Soc. Jpn. Ser. II* **93**, 5–48 (2015).
54. M. Kanamitsu, W. Ebisuzaki, J. Woollen, S.-K. Yang, J. J. Hnilo, M. Fiorino, G. L. Potter, NCEP–DOE AMIP-II Reanalysis (R-2). *Bull. Am. Meteorol. Soc.* **83**, 1631–1644 (2002).
55. C. H. O'Reilly, T. Woolings, J. Zanna, A. Weisheimer, The impact of tropical precipitation on summertime Euro-Atlantic circulation via a circumglobal wave train. *J. Climate* **31**, 6483–6504 (2018).
56. E. M. Knudsen, Y. J. Orsolini, T. Furevik, K. I. Hodges, Observed anomalous atmospheric patterns in summers of unusual Arctic sea ice melt. *J. Geophys. Res. Atmos.* **120**, 2595–2611 (2015).
57. H. W. Linderholm, T. Ou, J.-H. Jeong, C. K. Folland, D. Gong, H. Liu, Y. Liu, D. Chen, Interannual teleconnections between the Summer North Atlantic Oscillation and the East Asian summer monsoon. *J. Geophys. Res. Atmos.* **116**, D13107 (2011).
58. E. W. Blockley, K. A. Peterson, Improving Met Office seasonal predictions of Arctic sea ice using assimilation of CryoSat-2 thickness. *Cryosphere* **12**, 3419–3438 (2018).
59. K. Strommen, S. Juricke, F. Cooper, Improved teleconnection between Arctic sea ice and the North Atlantic Oscillation through stochastic process representation. *Weather Clim. Dynam.* **3**, 951–975 (2022).
60. T. Koenigk, U. Mikolajewicz, J. H. Jungclaus, A. Kroll, Sea ice in the Barents Sea: Seasonal to interannual variability and climate feedbacks in a global coupled model. *Clim. Dyn.* **32**, 1119–1138 (2009).
61. R. Alkama, E. N. Koffi, S. J. Vavrus, T. Diehl, J. A. Francis, J. Stroeve, G. Forzieri, T. Vihma, A. Cescatti, Wind amplifies the polar sea ice retreat. *Environ. Res. Lett.* **15**, 124022 (2020).
62. D. M. Smith, J. A. Screen, C. Deser, J. Cohen, J. C. Fyfe, J. García-Serrano, T. Jung, V. Kattsov, D. Matei, R. Msadek, Y. Peings, M. Sigmond, J. Ukita, J.-H. Yoon, X. Zhang, The Polar Amplification Model Intercomparison Project (PAMIP) contribution to CMIP6: Investigating the causes and consequences of polar amplification. *Geosci. Model Dev.* **12**, 1139–1164 (2019).
63. Y. Kushnir, Interdecadal variations in North Atlantic sea surface temperature and associated atmospheric conditions. *J. Climate* **7**, 141–157 (1994).
64. H. S. Baker, T. Woollings, C. E. F. Forest, M. R. Allen, The linear sensitivity of the North Atlantic Oscillation and eddy-driven jet to SSTs. *J. Climate* **32**, 6491–6511 (2019).
65. D. P. Rowell, C. K. Folland, K. Maskell, J. A. Owen, Modelling the influence of global sea surface temperatures on the variability and predictability of seasonal Sahel rainfall. *Geophys. Res. Lett.* **19**, 905–908 (1992).
66. H. Douville, S. Bielli, C. Cassou, M. Déqué, N. M. J. Hall, S. Tyteca, A. Voldoire, Tropical influence on boreal summer mid-latitude stationary waves. *Clim. Dyn.* **37**, 1783–1798 (2011).
67. A. A. Scaife, R. E. Comer, N. J. Dunstone, J. R. Knight, D. M. Smith, C. MacLachlan, N. Martin, K. A. Peterson, D. Rowlands, E. B. Carroll, S. Belcher, J. Slingo, Tropical rainfall, Rossby waves and regional winter climate predictions. *Q. J. R. Meteorol. Soc.* **143**, 1–11 (2017).
68. P. Y. F. Siew, C. Li, M. Ting, S. P. Sobolowski, Y. Wu, X. Chen, North Atlantic Oscillation in winter is largely insensitive to autumn Barents-Kara sea ice variability. *Sci. Adv.* **7**, eabg4893 (2021).
69. Y. Nie, Y. Zhang, X.-Q. Yang, H.-L. Ren, Winter and summer Rossby wave sources in the CMIP5 models. *Earth Space Sci.* **6**, 1831–1846 (2019).
70. S. Hu, T. Zhou, B. Wu, The physical processes dominating the impact of the summer North Atlantic Oscillation on the Eastern Tibetan Plateau summer rainfall. *J. Climate* **35**, 4077–4090 (2022).
71. I. N. James, *Introduction to Circulating Atmospheres* (Cambridge Univ. Press, first paperback edition, 1995), pp. 422.
72. A. A. Scaife, D. Smith, A signal-to-noise paradox in climate science. *npj Clim. Atmos. Sci.* **1**, 28 (2018).
73. C. Strong, G. Magnusdottir, Dependence of NAO variability on coupling with sea ice. *Clim. Dyn.* **36**, 1681–1689 (2011).
74. M. Mori, Y. Kosaka, M. Watanabe, B. Taguchi, H. Nakamura, M. Kimoto, Reply to: Is sea-ice-driven Eurasian cooling too weak in models? *Nat. Clim. Change* **9**, 937–939 (2019).
75. J. J. Barsugli, D. S. Battisti, The basic effects of atmosphere–ocean thermal coupling on midlatitude variability. *J. Atmos. Sci.* **55**, 477–493 (1998).
76. T. Woollings, C. Li, M. Drouard, E. Dunn-Sigouin, K. A. Elmestekawy, M. Hell, B. Hoskins, C. Mbengue, M. Patterson, T. Spengler, The role of Rossby waves in polar weather and climate. *Weather Clim. Dynam.* **4**, 61–80 (2023).
77. R. Blackport, J. A. Screen, Observed statistical connections overestimate the causal effects of Arctic sea ice changes on midlatitude winter climate. *J. Climate* **34**, 3021–3038 (2021).
78. Z. Liu, C. Risi, F. Codron, Z. Jian, Z. Wei, X. He, C. J. Poulsen, Y. Wang, D. Chen, W. Ma, Y. Cheng, G. J. Bowen, Atmospheric forcing dominates winter Barents-Kara sea ice variability on interannual to decadal time scales. *Proc. Natl. Acad. Sci. U.S.A.* **119**, e2120770119 (2022).
79. N. Dunstone, D. M. Smith, S. C. Hardiman, L. Hermanson, S. Ineson, G. Kay, C. Li, J. F. Lockwood, A. A. Scaife, H. Thornton, M. Ting, L. Wang, Skilful predictions of the Summer North Atlantic Oscillation. *Nat. Commun. Earth Environ.* **4**, 409 (2023).
80. T. Chronis, D. E. Raitsos, D. Kassis, A. Sarantopoulo, The Summer North Atlantic Oscillation Influence on the Eastern Mediterranean. *J. Climate* **24**, 5584–5596 (2011).
81. I. B. , B. Liebmann, D. Fortuny, G. J. van Oldenborgh, Observed and simulated impacts of the summer NAO in Europe: Implications for projected drying in the Mediterranean region. *Clim. Dyn.* **39**, 709–727 (2012).
82. M. J. Barcikowska, S. B. Kapnick, L. Krishnamurty, S. Russo, A. Cherchi, C. K. Folland, Changes in the future summer Mediterranean climate: Contribution of teleconnections and local factors. *Earth Syst. Dyn.* **11**, 161–181 (2020).
83. M. Gaetani, B. Pohl, H. Douville, B. Fontaine, West African Monsoon influence on the summer Euro-Atlantic circulation. *Geophys. Res. Lett.* **38**, L09705 (2011).
84. J. A. Lowe, D. Bernie, P. Bett, L.Bricheno, S. Brown, D. Calvert, R. Clark, K. Eagle, T. Edwards, G. Fosser, F. Fung, L. Gohar, P. Good, J. Gregory, G. Harris, T, Howard, N. Kaye, E. Kendon, J. Krijnen, P. Maisey, R. McDonald, R. McInnes, C. McSweeney, J. F.B. Mitchell, J. Murphy, M. Palmer, C. Roberts, J. Rostron, D. Sexton, H. Thornton, J. Tinker, S. Tucker, K, Yamazaki, S. Belcher, *UKCP18 Science Overview Report* (Met Office, 2019).
85. K. van der Wiel, T. J. Batelaan, N. Wanders, Large increases of multi-year droughts in north-western Europe in a warmer climate. *Clim. Dyn.* **60**, 1781–1800 (2023).
86. L. C. Slivinski, G. P. Compo, J. S. Whitaker, P. D. Sardeshmukh, B. S. Giese, C. McColl, R. Allan, X. Yin, R. Vose, H. Titchner, J. Kennedy, L. J. Spencer, L. Ashcroft, S. Brönnimann, M. Brunet, D. Camuffo, R. Cornes, T. A. Cram, R. Crouthamel, F. Domínguez-Castro, J. E. Freeman, J. Gergis, E. Hawkins, P. D. Jones, S. Jourdain, A. Kaplan, H. Kubota, F. Le Blancq, T.-C. Lee, A. Lorrey, J. Luterbacher, M. Maugeri, C. J. Mock, G. W. K. Moore, R. Przybylak, C. Pudmenzky, C. Reason, V. C. Slonosky, C. A. Smith, B. Tinz, B. Trewin, M. A. Valente, X. L. Wang, C. Wilkinson, K. Wood, P. Wyszyński, Towards a more reliable historical reanalysis: Improvements for version 3 of the twentieth century reanalysis system. *Q. J. R. Meterol. Soc.* **145**, 2876–2908 (2019).
87. E. Kalnay, M. Kanamitsu, R. Kistler, W. Collins, D. Deaven, L. Gandin, M. Iredell, S. Saha, G. White, J. Woollen, Y. Zhu, M. Chelliah, W. Ebisuzaki, W. Higgins, J. Janowiak, K. C. Mo,

C. Ropelewski, J. Wang, A. Leetmaa, R. Reynolds, R. Jenne, D. Joseph, The NCEP/NCAR 40-Year Reanalysis Project. *Bull. Amer. Meteor. Soc.* **77**, 437–471 (1996).
88. Y. Batrak, M. Müller, On the warm bias in atmospheric reanalyses induced by the missing snow over Arctic sea-ice. *Nat. Commun.* **10**, 4170 (2019).
89. I. T. Jolliffe, *Principal Component Analysis*, Springer Series in Statistics (Springer-Verlag, ed. 2, 2002), pp. 487.
90. C. S. Bretherton, C. Smith, J. M. Wallace, An intercomparison of methods for finding coupled patterns in climate data. *J. Climate* **5**, 541–560 (1992).
91. J. M. Wallace, C. Smith, C. S. Bretherton, Singular value decomposition of wintertime sea surface temperature and 500-mb height anomalies. *J. Climate* **6**, 561–576 (1992).
92. W. Ebisuzaki, A method to estimate the statistical significance of a correlation when the data are serially correlated. *J. Climate* **10**, 2147–2153 (1997).

Acknowledgments: We thank the KNMI Climate Explorer site (https://climexp.knmi.nl/start.cgi) for hosting the calculation of the maximum covariance analysis and some of the diagrams, including access to ERA5, and S. Ineson for providing some software and derived datasets. We also thank the reviewers for excellent comments that much improved this paper. **Funding:** J.K., A.A.S., and C.K.F. were supported by the Met Office Hadley Centre Climate Programme funded by BEIS and Defra. This work and its contributors (C.K.F. and A.A.S.) were partly funded by the Met Office Climate Science for Service Partnership (CSSP) China project under the International Science Partnerships Fund (ISPF). C.K.F. and H.W.L. were funded by Swedish Research Council contract grant number 2012-05246. T.O., H.W.L., and D.C. were funded by the Swedish Foundation for International Cooperation in Research and Higher Education (STINT) (CH2019-8377 and CH2020-8767) and the Swedish national strategic research program MERGE. The CMIP6 and ERA5 computations were funded by resources provided by the National Academic Infrastructure for Supercomputing in Sweden (NAISS) and the Swedish National Infrastructure for Computing (SNIC) at the National Supercomputer Centre in Sweden (NSC) partially funded by the Swedish Research Council through grant agreements no. 2022-06725 and no. 2018-05973. Part of the CMIP6 computations is conducted on the Analysis Platforms at DKRZ, which is part of the IS-ENES3 project that has received funding from the European Union's Horizon 2020 research and innovation program under grant agreement no. 824084. **Author contributions:** C.K.F. conceived and led the paper, mainly wrote it, and provided some of the diagrams. T.O. carried out all the CMIP6 and ERA5 calculations and associated diagrams and provided some of the text, while H.W.L., J.K., A.A.S., and D.C. also contributed to the text including interpretation of the results where A.A.S. particularly contributed to Discussion and H.W.L. to Introduction. **Competing interests:** The authors declare that they have no competing interests. **Data and materials availability:** All data needed to evaluate the conclusions in the paper are present in the paper and/or the Supplementary Materials. The locations of observed and model datasets used are shown in Materials and Methods by the relevant URLs.

Submitted 4 September 2023
Accepted 11 October 2024
Published 13 November 2024
10.1126/sciadv.adk6693
