# Supplementary material for: The Summer North Atlantic Oscillation, Arctic sea ice, and Arctic jet Rossby wave forcing
Source: Sci Adv. 2024 Nov 13;10(46):eadk6693. doi: 10.1126/sciadv.adk6693 (PMC11639673; doi:10.1126/sciadv.adk6693)
Supplement: Supplementary file 3 — Erratum published 1 August 2025: The original version of the Research Article included errors in Fig. 5 (B and E) and fig. S4B of the Supplementary Materials. In both figures, the authors intended to show the results of the average of JRA-55 and NCEP2; however, the figures show the average of ERA5, JRA-55, and NCEP2. The corresponding captions were incorrect as well, which originally stated that the figures showed the difference between high and low sea ice years. The figures, instead, show the average of all data. Corresponding text in the main text discussing Fig. 5 was also incorrect. In addition, the original version of the article included inadvertent typos related to the contents of Table 3. In the Table 3 caption and corresponding text in Results, “negative sign of the SNAO” should say “positive sign of the SNAO.” The following corrections have been implemented: • Figure 5 has been replaced with the corrected figure, and its caption has been updated. • The Supplementary Materials PDF has been replaced with a corrected fig. S4B and caption. • Text in the Results has been updated to reflect the corrections to Fig. 5. • Text in the Results and Table 3 title have been updated to fix the typo. The authors’ conclusions are not affected by these corrections. The full text and PDF versions of the article and the Supplementary Materials PDF have been updated. The original version of the Supplementary Materials is available here: [file sciadv.adk6693_sm.v1.pdf]

Science Advances
AAAS

Supplementary Materials for

The Summer North Atlantic Oscillation, Arctic sea ice, and Arctic jet Rossby wave forcing

Chris K. Folland *et al.*

Corresponding author: Chris K. Folland, chris.folland@metoffice.gov.uk

Sci. Adv. **10**, eadk6693 (2024)
DOI: 10.1126/sciadv.adk6693

This PDF file includes:

Figs. S1 to S9
Tables S1 to S4

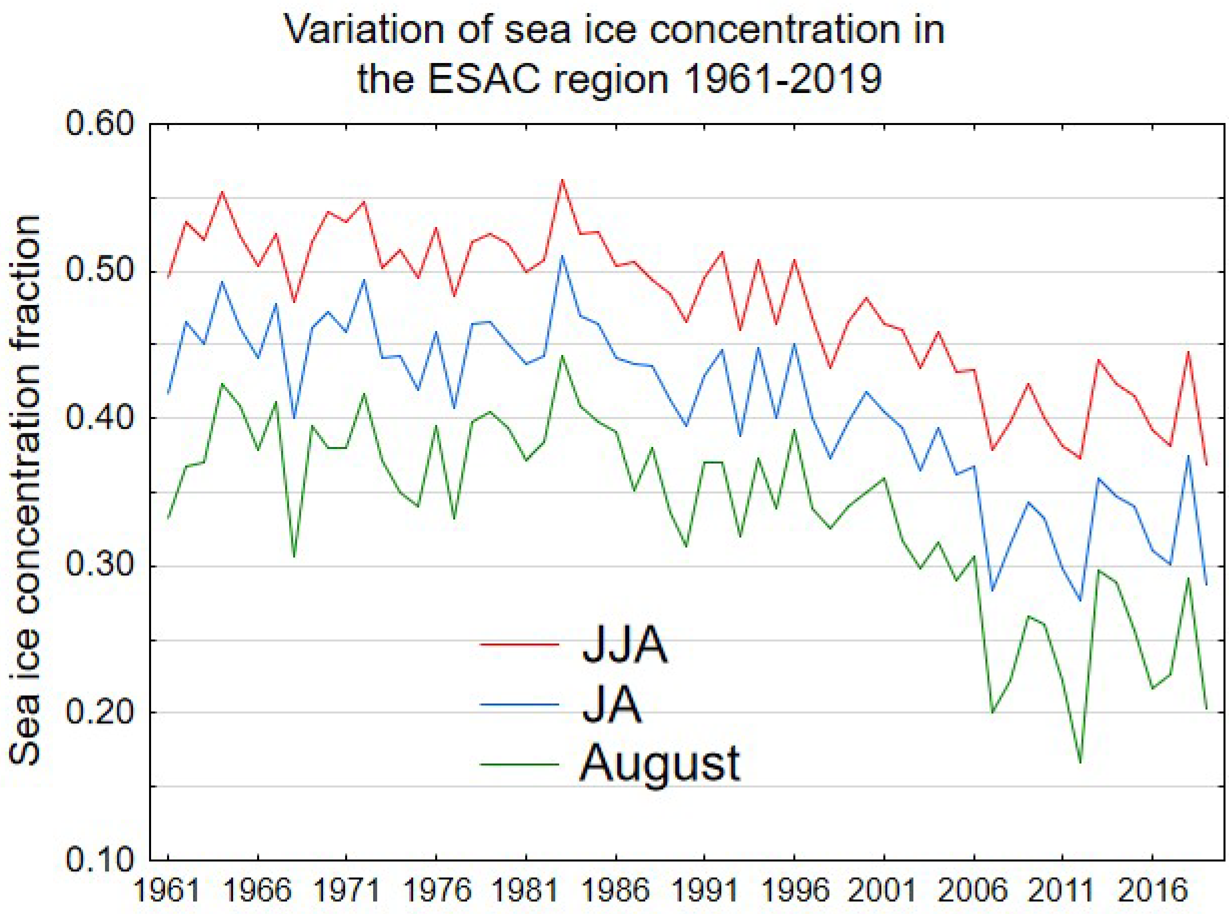

Fig. S1. Variation of the sea ice concentration (SIC) fraction in the East Siberian- to Arctic Canada (ESAC) region averaged over June-July-August (JJA), July-August (JA) and August, 1961-2019.

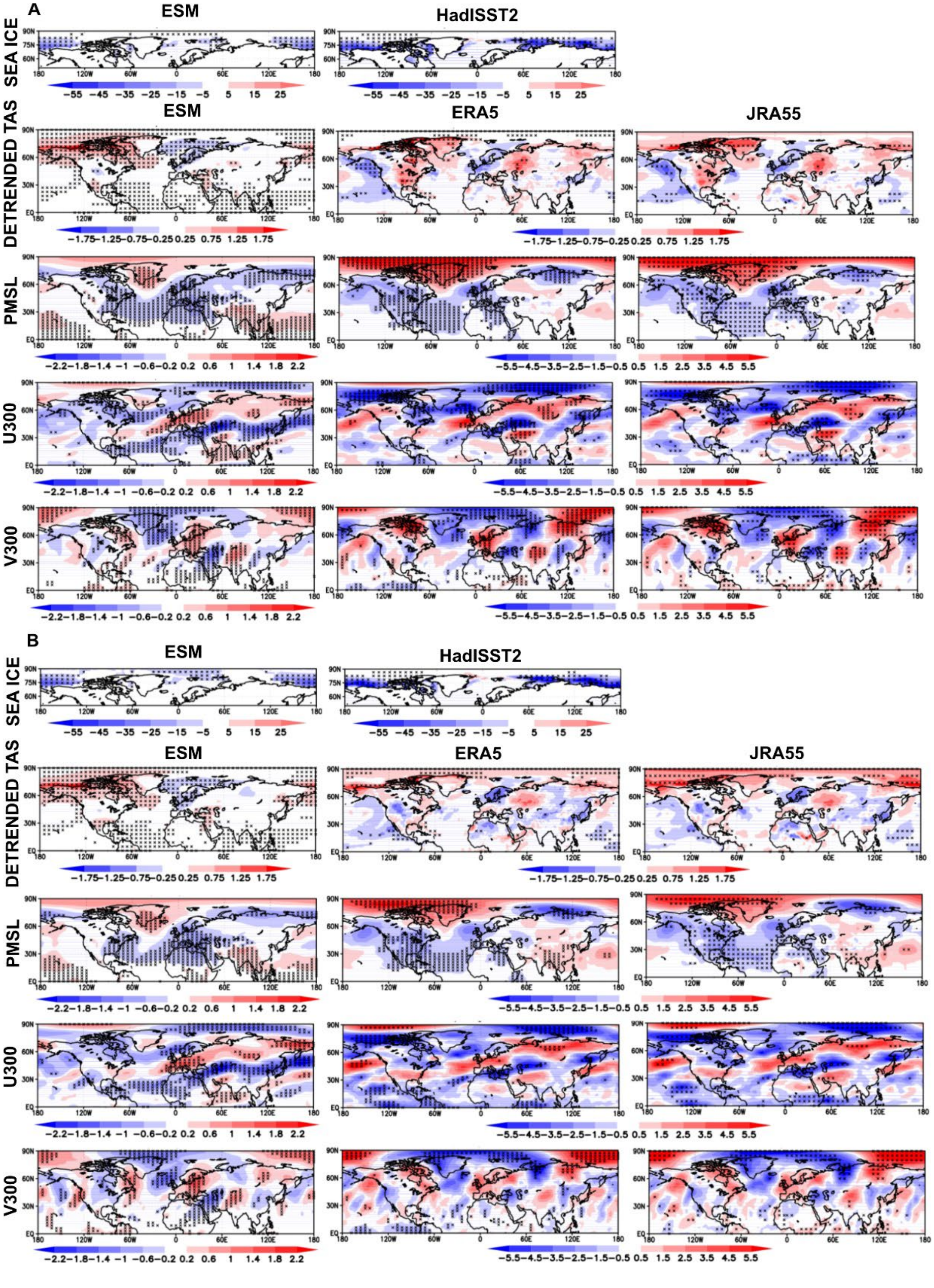

Fig. S2. **Differences in key climate variables for Coupled Model Intercomparison Project Phase 6 (CMIP6) models and ERA5 and JRA-55 observations (except for HadISST2 sea**

ice) over the Northern Hemisphere between the 8 lowest ESAC sea ice concentration years and 8 highest ESAC sea ice concentration years.
A) JA, 1979-2015. ESM denotes the ensemble mean difference from the 12 models. Black crosses represent significance at the 5 % level using a two-sided t test. (B) as for (A) but August.

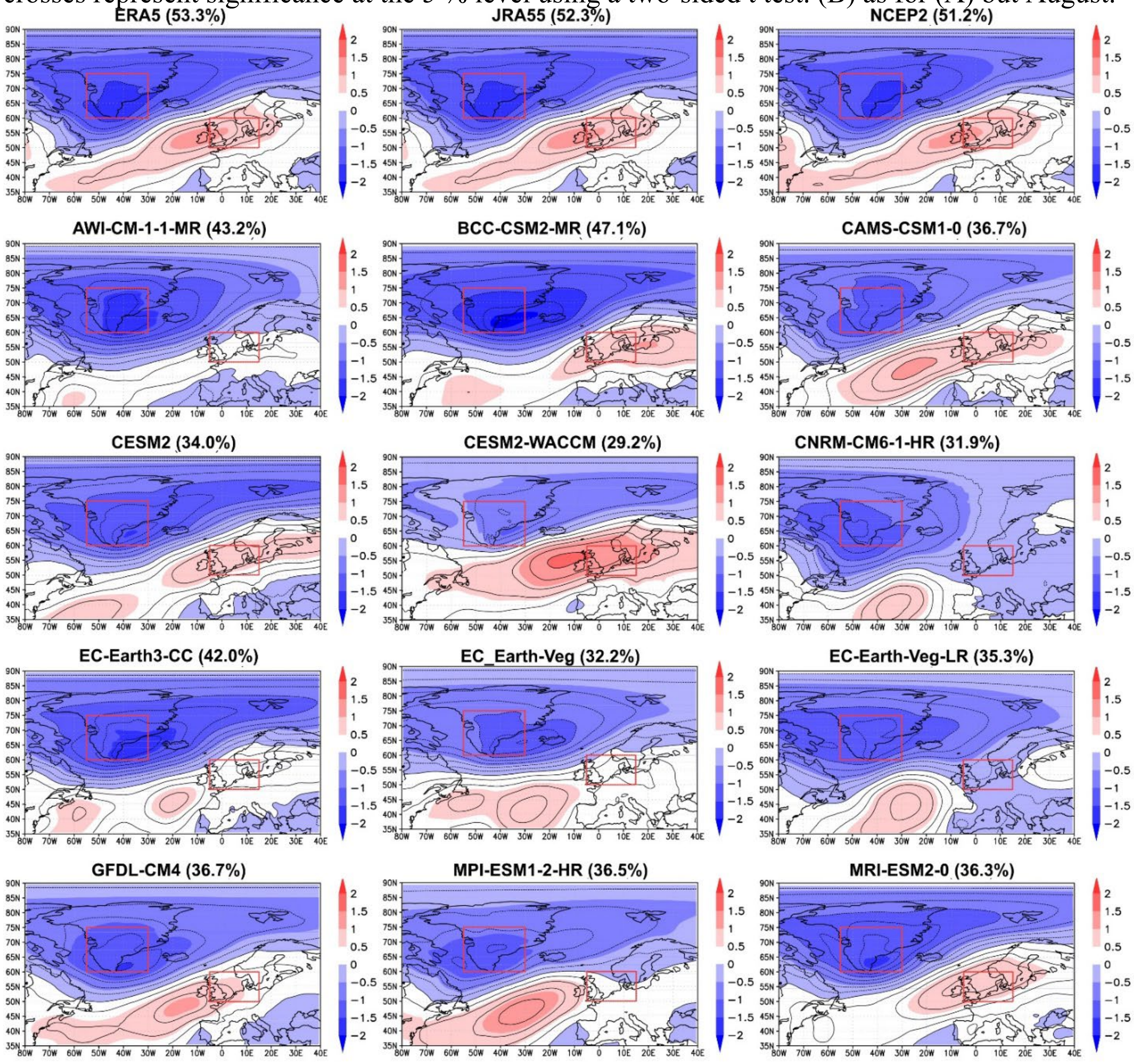

Fig. S3. The first covariance eigenvector (EOF1) representing the Summer North Atlantic Oscillation (SNAO) (with fraction of total explained covariance) of JJA pressure at mean sea level for ERA5, JRA-55, and NCEP2, and the 12 selected coupled climate models during 1979-2015. The two red squares show the regions used to calculate the regional SNAO index (([55ºW-30ºW, 60-75ºN] – [5ºW-15ºE, 50 -60ºN]).

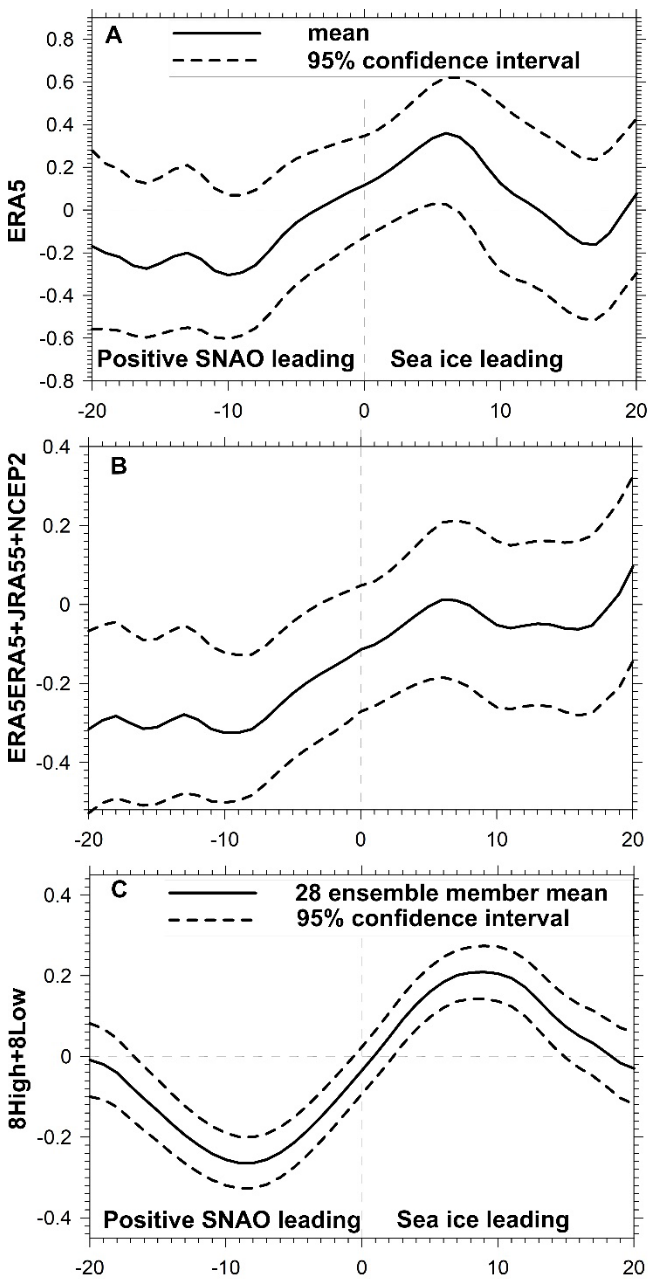

Fig. S4. 15-day running mean lead and lag correlations between the SNAO index and ESAC SIC in reanalysis and CMIP6 models for August. (**A**) As shown by ERA5. (**B**) As shown by the average of JRA-55 and NCEP2. (**C**) As shown by the difference between the extreme low 8 SIC years and the extreme high SIC years. The x-axis scale is that of SIC lags and leads compared to the SNAO, shown lagging by up to 20 days (left) and leading by up to 20 days (right). The SNAO index is calculated based on the regional difference version (([55°W-30°W, 60-75°N] – [5°W-15°E, 50 -60°N]). The seasonal cycle of the daily time series has been removed.

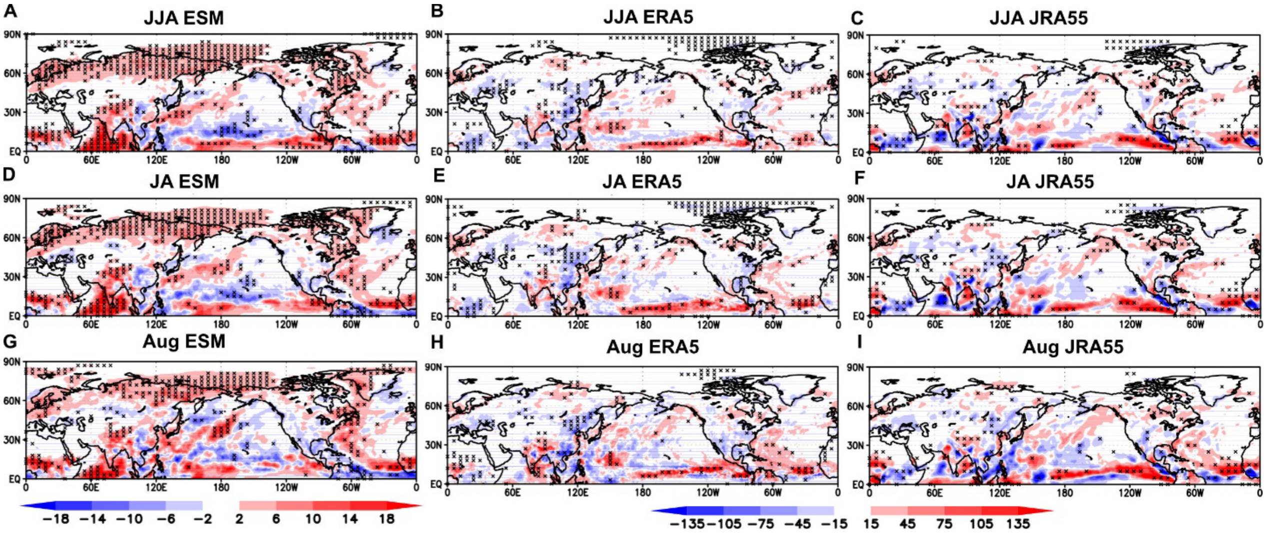

Fig. S5. Difference in Northern hemisphere precipitation (mm) for the 8 extreme pairs of ESAC SIC years, here low SIC minus high SIC. (**A**) For CMIP6 JJA, (**B**) For ERA5 JJA, (**C**) For JJA JRA-55, (**D**) For CMIP6 JA, (**E**) For ERA5 JA, (**F**) For JA JRA-55, (**G**) For CMIP6 August, (**H**) For ERA5 August, (**I**) For JRA-55 August**.** Note enhanced precipitation over the southern node of the SNAO region around UK and northwest Europe and reduced precipitation over the northern node in the ESAC region in both models and observations, corresponding to the negative SNAO state. Black crosses represent significance at the 5% level using a two-sided t test.

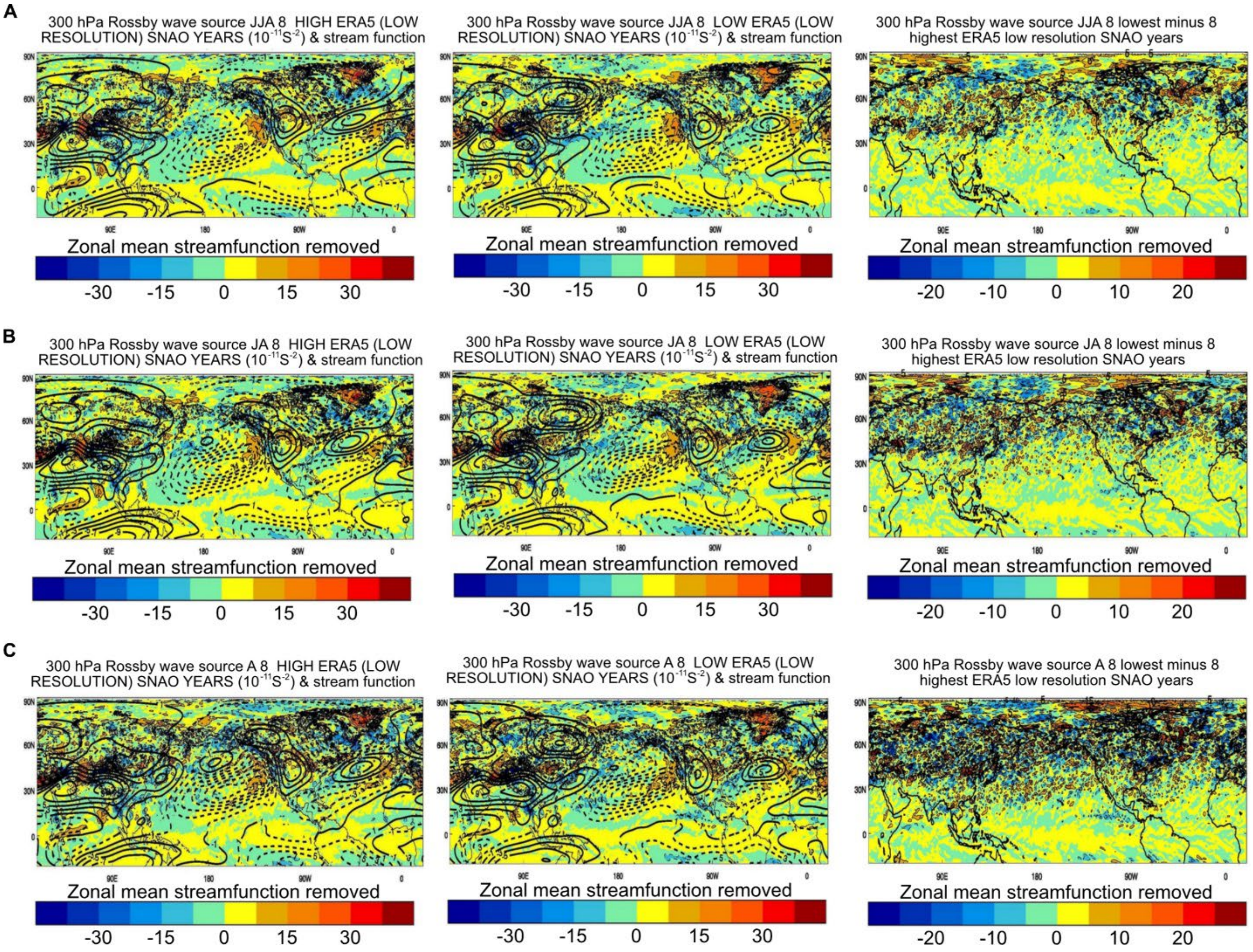

Fig. S6. Rossby wave source diagnostics at 300hPa between the 8 lowest SNAO years and the 8 highest SNAO years, 1979-2015, for ERA5 at a resolution of 0.75°x0.75°. (**A**) First column JJA lowest 8 SNAO years. Second column. JJA highest 8 SNAO years, third column the differences in JJA between them. (**B**) JA as for (A), (**C**) August as for (A). Relative sources are red and yellow, sinks are green and blue. Units are 10^{-11} s^{-2}. Includes the tropics to 20°S. Positive (solid lines) and negative (dashed lines) stream function contours are shown for the 8 lowest and 8 highest years where the zonal mean stream function has been removed. No significance is shown for the noisy Rossby wave diagnostics but see Table 3.

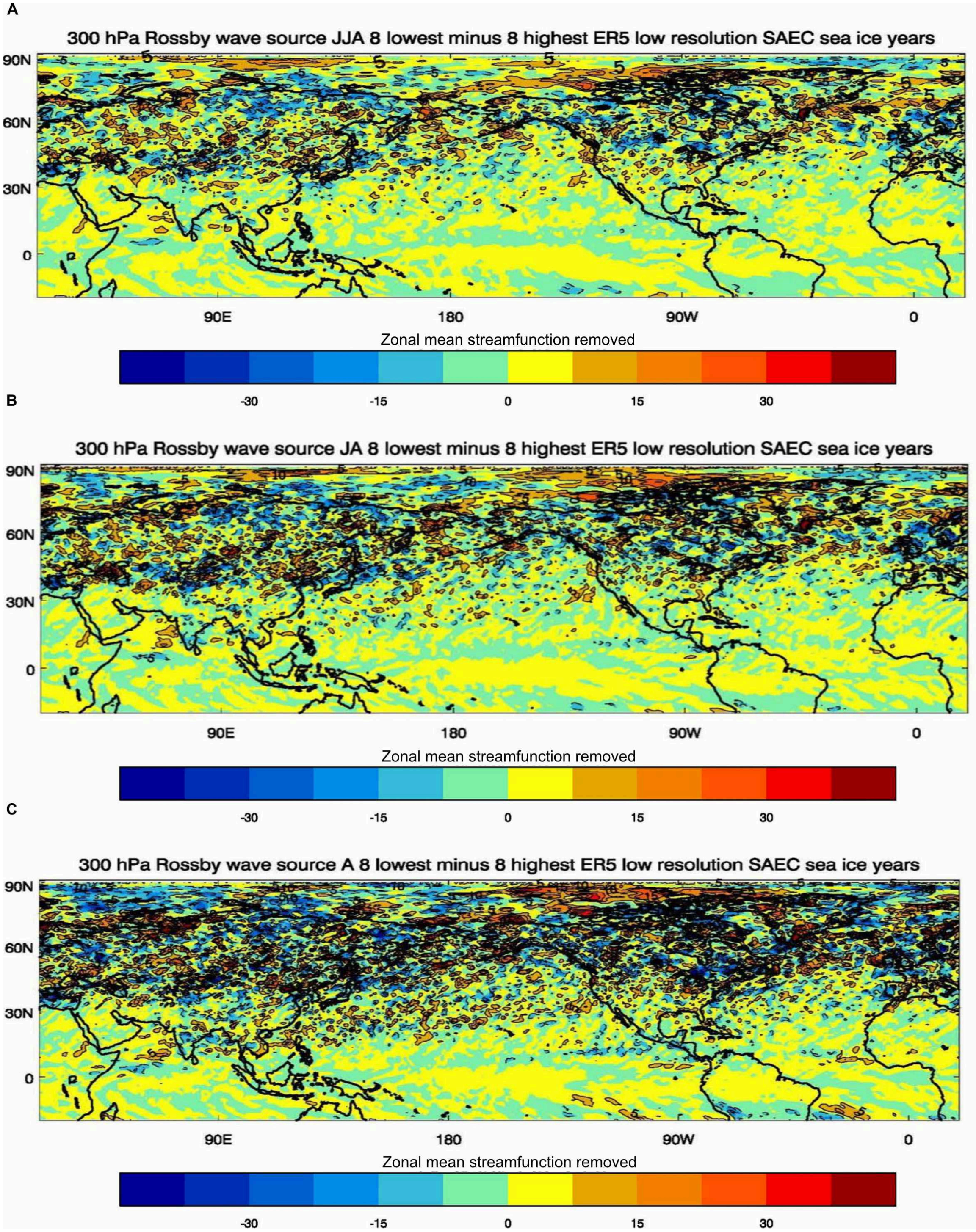

Fig. S7. Rossby wave source diagnostic differences at 300hPa between the 8 SIC years and the 8 highest years, 1979-2015 for ERA5 at a resolution of 0.75°x0.75° shown on a larger scale. (**A**) For JJA (**B**) For JA (**C**) For August. Relative sources are red and yellow, sinks are green and blue. Units are 10^{-11} s^{-2}. Includes the tropics to 20°S. The Zonal mean stream function has been removed. No significance is shown for these noisy diagnostics.

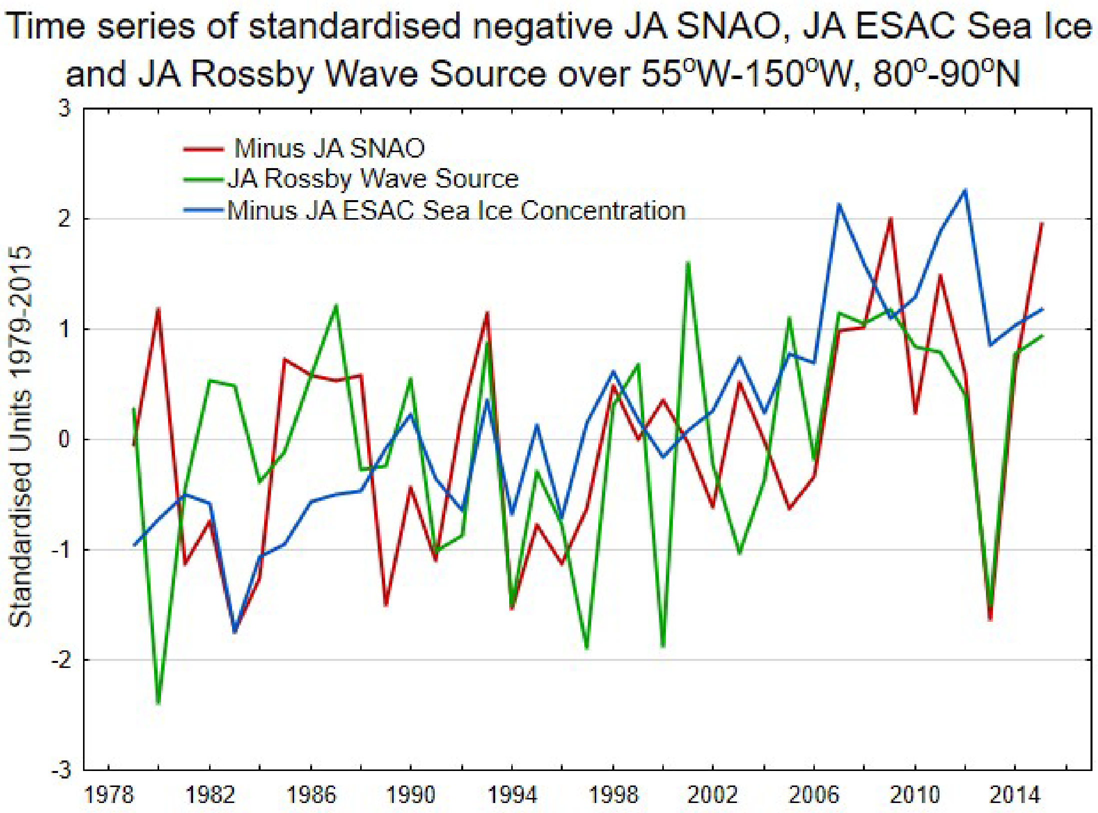

Fig. S8. Time series of the negative of the standardized JA SIC over the ESAC region, the negative JA SNAO and JA Rossby wave source. All data are standardised over 1979-2015. Higher Rossby Wave source values are more positive. Anything less than 0.29 standard deviations of the Rossby Wave Source has a negative absolute value, making it a Rossby Wave sink.

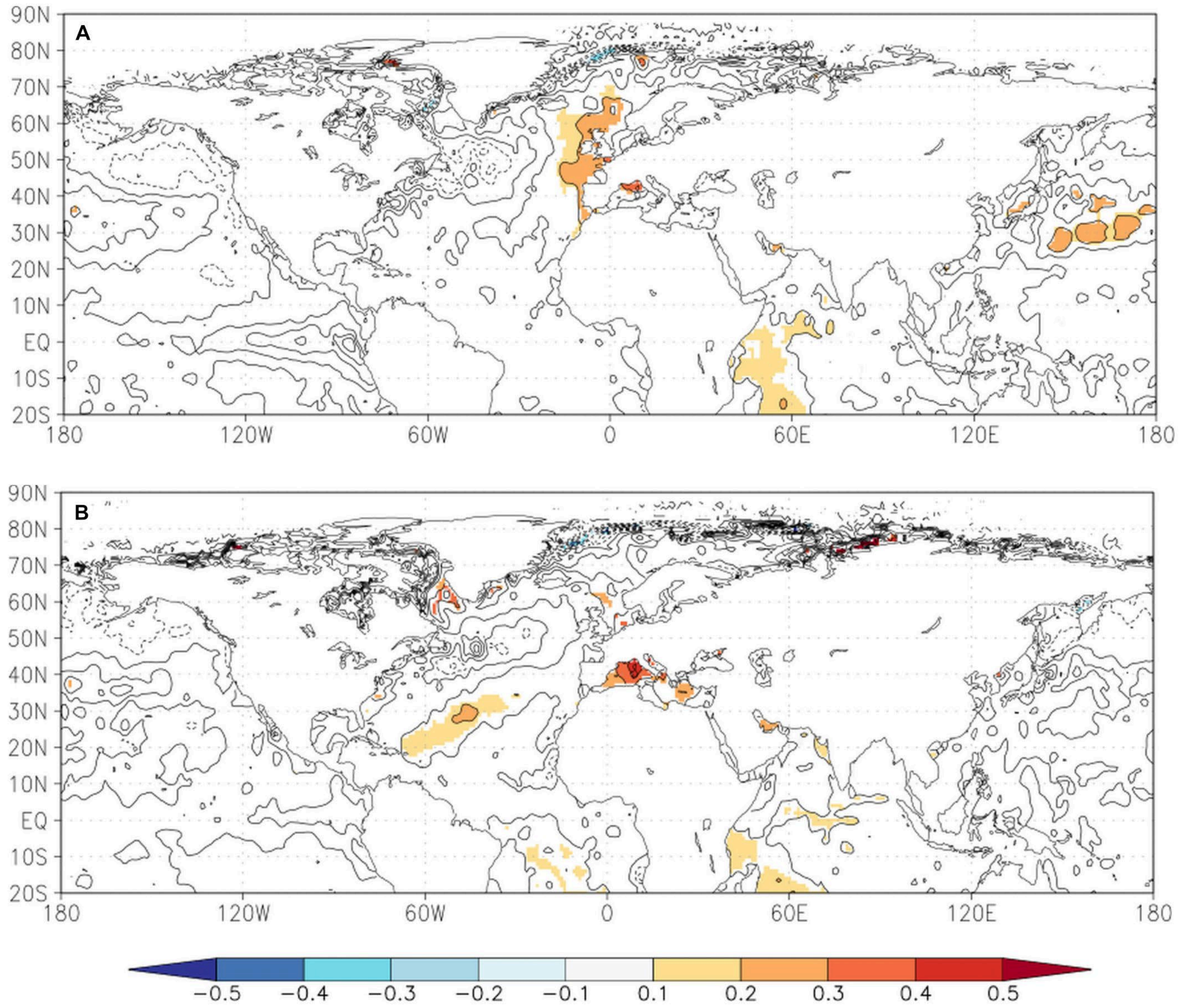

Fig. S9. Regression of the SNAO against HadISST1 sea surface temperature (SST), 1979-2015. (**A**) For JJA SNAO and the previous May SST. (**B**) As (A) but for the JA SNAO and the previous June SSTs. Areas significant at the 5% level are coloured.

Table S1. Covariance squared explained (%) by the first two pairs of singular value decomposition (SVD) patterns of sea ice concentration (SIC) and pressure at mean sea level (PMSL) in June-July-August (JJA), July-August (JA) and August over 1979-2015

	JJA	JA	August
SVD1	42.0	37.0	28.6
SVD2	10.4	11.0	10.1

Table S2. Mean difference of the normalized regional Summer North Atlantic Oscillation (SNAO) index (the difference between the two SNAO regional nodes ((55°W-30°W, 60-75°N) – (5°W-15°E, 50 -60°N)),) for the 8 lowest minus 8 highest SIC years, averaged for 12 global climate models (GCMs) (ESM12) and ERA 5 and JRA-55. The regional SNAO index is initially calculated separately for each GCM. One star represents significance at the 5% level, two stars at the 1% level.

	ESM12	ERA5	JRA-55
Aug	-0.27**	-0.77	-0.76
JA	-0.38**	-1.48*	-1.44*
JJA	-0.36**	-1.45*	-1.41*

Table S3. Ranking and tests of the 37 candidate CMIP6 models used to select the best twelve for studying the relationship between the SNAO and ESAC SIC. R-JJA and R-JA are correlations between modelled and observed PMSL over the SNAO region in 1979-2015 and RMSE-JJA and RMSE-JA are root mean squared errors (hPa).

Rank	R-JJA	RMSE-JJA	R-JA	RMSE-JA	CMIP6 CGCM
1	0.98	0.93	0.97	1.07	AWI-CM-1-1-MR
2	0.97	1.08	0.96	1.17	EC-Earth3-Veg-LR
3	0.97	1.4	0.97	1.58	BCC-CSM2-MR
4	0.96	1.31	0.96	1.35	EC-Earth3-Veg
5	0.96	1.21	0.95	1.41	EC-Earth3-CC
6	0.96	1.68	0.97	1.68	CAMS-CSM1-0
7	0.96	1.76	0.96	1.87	MRI-ESM2-0
8	0.94	1.76	0.94	2.1	CESM2
9	0.94	1.82	0.94	1.82	MPI-ESM1-2-HR
10	0.94	1.79	0.94	2.17	CESM2-WACCM
11	0.90	1.61	0.92	1.57	GFDL-CM4
12	0.92	1.85	0.94	1.66	CNRM-CM6-1-HR
13	0.89	1.7	0.92	1.61	GFDL-ESM4
14	0.90	1.61	0.91	1.74	FGOALS-f3-L
15	0.92	2.64	0.95	2.2	CIESM
16	0.90	1.71	0.91	1.72	NESM3
17	0.93	2.37	0.93	2.43	CMCC-CM2-SR5
18	0.93	2.2	0.93	2.6	NorESM2-MM

19	0.92	2.43	0.92	2.41	MPI-ESM1-2-LR
20	0.91	2.14	0.91	2.42	NorESM2-LM
21	0.92	2.74	0.94	2.64	CMCC-ESM2
22	0.91	2.53	0.91	2.58	ACCESS-ESM1-5
23	0.87	1.88	0.86	2.1	GISS-E2-1-G
24	0.91	2.62	0.91	2.66	FIO-ESM-2-0
25	0.90	2.23	0.89	2.82	CanESM5
26	0.88	2.93	0.92	2.63	HadGEM3-GC31-LL
27	0.88	2.32	0.87	2.64	IPSL-CM6A-LR
28	0.89	2.35	0.88	2.87	CanESM5-CanOE
29	0.74	2.87	0.78	2.88	MIROC6
30	0.77	2.93	0.78	2.89	MIROC-ES2L
31	0.82	3.22	0.82	3.32	UKESM1-0-LL
32	0.77	3.01	0.75	3.22	INM-CM4-8
33	0.69	3.44	0.8	3.07	CNRM-ESM2-1
34	0.79	3.68	0.81	3.71	TaiESM1
35	0.77	4.37	0.83	4.33	ACCESS-CM2
36	0.76	3.17	0.75	3.45	INM-CM5-0
37	0.63	3.66	0.74	3.24	CNRM-CM6-1

Table S4. Ranking and evaluation statistics (Correlation R and root mean square error (RMSE) (hPa)) of the 36 candidate CMIP6 AMIP models used to select the best twelve for studying the possible response of atmospheric and land components to prescribed sea surface temperatures (SSTs) and SICs.

Rank	R-JJA	RMSE-JJA	R-JA	RMSE-JA	CMIP6 GCM
1	0.98	1.3	0.98	1.3	CNRM-CM6-1-HR
2	0.97	1.1	0.97	1.1	MPI-ESM1-2-HR
3	0.96	1.5	0.97	1.4	CIESM
4	0.96	1.1	0.96	1.4	EC-Earth3-Veg
5	0.95	1.3	0.96	1.5	EC-Earth3
6	0.94	1.4	0.94	1.5	CAMS-CSM1-0
7	0.94	1.4	0.94	1.6	GFDL-ESM4
8	0.94	1.6	0.95	1.6	BCC-ESM1
9	0.95	1.7	0.96	1.7	HadGEM3-GC31-MM
10	0.95	1.7	0.95	1.9	MPI-ESM1-2-LR
11	0.98	2.4	0.98	2.4	FGOALS-f3-L
12	0.93	1.6	0.91	1.8	GISS-E2-2-G
13	0.94	2.0	0.95	2.5	E3SM-1-0
14	0.97	2.5	0.97	2.8	BCC-CSM2-MR
15	0.94	2.4	0.95	2.3	CESM2-WACCM
16	0.91	1.6	0.92	1.7	GFDL-CM4
17	0.93	1.6	0.92	2.1	CanESM5
18	0.93	2.0	0.93	2.3	ACCESS-ESM1-5

19	0.91	2.0	0.93	1.9	CMCC-CM2-SR5
20	0.92	2.2	0.93	2.1	CESM2-WACCM-FV2
21	0.91	1.6	0.89	1.9	GISS-E2-1-G
22	0.91	2.4	0.93	2.3	HadGEM3-GC31-LL
23	0.91	2.5	0.93	2.5	CESM2-FV2
24	0.93	2.8	0.94	2.9	CESM2
25	0.89	2.0	0.88	2.2	IPSL-CM6A-LR
26	0.90	2.4	0.89	2.5	FIO-ESM-2-0
27	0.87	2.5	0.89	2.5	KACE-1-0-G
28	0.88	2.7	0.89	2.8	ACCESS-CM2
29	0.87	2.6	0.87	2.5	MIROC6
30	0.89	2.8	0.88	3.0	IITM-ESM
31	0.87	3.4	0.88	3.3	KIOST-ESM
32	0.82	2.7	0.78	2.9	INM-CM5-0
33	0.86	2.9	0.85	3.1	MPI-ESM-1-2-HAM
34	0.78	2.8	0.74	3.2	INM-CM4-8
35	0.78	3.8	0.85	3.6	CNRM-CM6-1
36	0.77	3.9	0.85	3.7	CNRM-ESM2-1
